# MetaMP Ecosystem for Unified, Auditable, and Benchmark-Ready Data for Reliable Membrane Protein Annotation

**DOI:** 10.34133/csbj.0165

**Published:** 2026-08-20

**Authors:** Ebenezer Awotoro, Chisom Anyabolu, Florian Schwarz, Johannes Tauscher, Dominik Heider, Katharina Ladewig, Christel Le Bon, Karine Moncoq, Bruno Miroux, Georges Hattab

**Affiliations:** ^1^ Center for Artificial Intelligence in Public Health Research (ZKI-PH), Robert Koch Institute, Berlin 13353, Germany.; ^2^ Department of Mathematics and Computer Science, University of Marburg, Marburg, Germany.; ^3^ Institute of Medical Informatics, University of Münster, Münster, Germany.; ^4^ Université Paris Cité, Centre National de la Recherche Scientifique (CNRS), Biochimie des Protéines Membranaires, UMR7099 Paris, France.; ^5^ Department of Mathematics and Computer Science, Freie Universität Berlin, Berlin 14195, Germany.

## Abstract

Experimentally resolved membrane-protein structures have increased substantially, yet annotations remain fragmented across resources differing in scope, curation criteria, and identifier conventions, complicating cross-database comparison and downstream analysis. We present MetaMP, a membrane-protein reconciliation and benchmarking platform that harmonizes metadata from MPstruc, RCSB PDB, OPM, and UniProt into a unified, searchable resource integrating 4,089 unique structures. MetaMP provides provenance-aware discrepancy analysis, quality-control workflows, and 2 assistive modules as reproducible baselines: (a) a broad structural-group classifier trained on OPM-derived membrane-orientation descriptors and (b) a transmembrane-segment benchmarking layer integrating sequence-based and structure-derived topology sources. Cross-source comparison identified 121 broad-group conflicts between MPstruc and OPM (2.96% of 4,089 harmonized entries). These contested cases were expert-reviewed to form a 121-record discrepancy benchmark. Under strict label matching, OPM agreed with expert annotations for 96 of 121 records (79.34%), while the MetaMP assistive classifier agreed for 25 of 121 (20.66%) and MPstruc for 17 of 121 (14.05%). Under benchmark-aware evaluation applying a biologically motivated label-collapsing rule, agreement reached 88.43% for MetaMP, 80.99% for OPM, and 78.51% for MPstruc. In a 24-participant task-oriented user study, structured training was associated with faster task completion, and the adapted SUS-style usability score averaged 72.81, placing the system in the above-average to good range. MetaMP is not intended to replace primary databases, but to make disagreement among them explicit, traceable, and biologically interpretable, providing a reproducible framework for annotation harmonization, expert-guided curation, and membrane-protein benchmarking. Source code and deployment materials are available at https://github.com/Ebenco36/MetaMP-Server.

## Introduction

Membrane proteins (MPs) are central to cellular life, mediating signal transduction, transport, catalysis, and membrane organization, and they account for a disproportionately large share of current therapeutic targets [1,2]. Broadly, MPs include integral proteins embedded in the lipid bilayer and peripheral proteins that associate with membrane surfaces without traversing it [3,4]. Their diversity and functional importance have made them a sustained focus of structural biology, computational biology, and drug discovery [4,5]. Since the first MP structure was reported in 1985 [6], advances in purification, stabilization, and structure-determination techniques such as cryo-electron microscopy, x-ray crystallography, and nuclear magnetic resonance (NMR) have substantially expanded the number and diversity of experimentally resolved MP structures in the Protein Data Bank and in specialized resources such as MPstruc [7–9]. Despite this growth, obtaining MPs in stable, functional form remains technically demanding. These experimental constraints continue to limit throughput, reproducibility, and systematic large-scale analysis [10–16].

In parallel, the MP data landscape has become increasingly fragmented as the number of curated resources has grown. Relevant annotations are distributed across databases that differ in scope, curation strategy, metadata structure, update frequency, and labeling conventions. As a result, researchers routinely encounter missing values, sparse fields, outdated identifiers, inconsistent topology labels, and discrepancies in structural classification. Constructing a reliable MP dataset therefore requires substantial preprocessing, identifier remapping, and manual reconciliation, and these steps are rarely documented in a way that is reproducible across research groups. These limitations underscore the need for a resource that not only aggregates information from multiple MP databases but also harmonizes annotations, preserves provenance, exposes disagreements, and supports systematic quality control.

Several existing resources address parts of this problem. Comparative analyses have highlighted substantial differences among MPstruc, OPM, TCDB, PDBTM, and related databases in coverage, annotation criteria, structural classification, and transmembrane-domain assignment [8,17–20]. UniTmp integrates transmembrane-protein annotations from several established resources into a unified view [19], and TMAlphaFold extends membrane-oriented interpretation to structures derived from AlphaFold models [21]. These resources are valuable and have significantly improved accessibility to MP annotations. However, their primary emphasis is on integration, annotation serving, or browsing, rather than on preserving source-specific disagreement, qualifying the benchmark suitability of individual entries, or enabling structured expert-guided reconciliation workflows. To our knowledge, no existing resource currently combines, within a single framework, provenance-preserving integration, systematic surfacing of cross-source disagreement, benchmark-suitability qualification, and structured expert-guided reconciliation support in a reproducible and auditable manner.

To address this gap, we developed MetaMP, an MP reconciliation and benchmarking platform that harmonizes metadata from MPstruc, RCSB PDB, OPM, and UniProt into a unified, provenance-aware, and interactive resource. MetaMP was designed not only to enrich MP metadata but also to support systematic comparison of annotations across databases, identify inconsistencies, and assist in expert review. Its emphasis is on complementing primary databases by making the disagreements among them explicit, traceable, and biologically interpretable. MetaMP introduces a shift from consensus-driven integration of database annotations to disagreement-aware reconciliation. The principal contribution of this work is a reproducible framework for harmonizing fragmented MP annotations and supporting expert-guided reconciliation. Specifically, MetaMP:

1. Unified, structure-aware integration: It harmonizes heterogeneous experimental and computational data into a standardized framework, making metadata accessible and searchable.
2. Immutable provenance and reproducibility: Traceability and reproducibility are supported through deterministic versioning and granular auditing of MP annotations.
3. Automated discrepancy and quality control: It automatically detects discrepancy candidates by identifying annotation conflicts and noncanonical configurations, surfacing scientific ambiguities for systematic expert review.
4. Benchmark-qualification infrastructure: It stratifies entries by confidence and assembles discrepancy-aware benchmark subsets. An assistive broad structural-group classifier and a transmembrane-segment benchmarking layer are provided as reproducible baselines to support expert prioritization rather than to replace biological judgment.
5. Coordinated analytical views: It provides analytical views that connect discrepancy review, topology comparison, exploratory analysis, and entry-level structural inspection.

To support evaluation on difficult real-world cases, we assembled a discrepancy-focused expert reference set of 121 PDB entries for which MPstruc and OPM disagreed in broad structural-group assignment, representing 2.96% of the 4,089 harmonized entries. These records cover transmembrane proteins (those whose polypeptide chain spans the lipid bilayer via α-helices or β-barrels), monotopic proteins (those that associate with only one leaflet of the membrane without traversal), and bitopic proteins (those spanning the bilayer via a single transmembrane segment). This benchmark was intentionally assembled from contested cases and is therefore suited for stress-testing reconciliation workflows rather than estimating disagreement prevalence across the full MP landscape. MetaMP is primarily designed for structural biologists, computational biologists, and database curators who face 4 concrete problems that existing primary databases do not currently address together: (a) assembling a benchmark dataset in which annotation confidence is explicitly qualified and contested entries are flagged rather than silently included; (b) triaging a set of candidate records for curation by identifying which entries carry cross-source disagreement and what its structural basis is; (c) conducting provenance-sensitive cross-database comparison in which the source of each annotation and its review state are visible alongside the annotation value; and (d) identifying entries where source databases disagree, understanding whether the disagreement is biological or technical in origin, and exporting the resolved subset for downstream use. For straightforward queries about membrane orientation or topology for a known protein, established primary databases such as OPM and MPstruc remain the most direct resources; MetaMP is not intended to replace them for those tasks.

The scientific value of these contributions depends critically on how annotation conflicts are treated throughout the pipeline. Rather than resolving inter-database disagreement through arbitrary or opaque reconciliation steps, MetaMP operates as a reconciliation layer that integrates annotations and systematically surfaces cases of conflict. Such conflicts are not uniformly distributed; they are enriched among biologically complex entries, including state-dependent proteins, multichain assemblies, and proteins with noncanonical membrane associations. In these instances, disagreement between databases frequently reflects substantive differences in structural state, curation criteria, or biological interpretation, rather than simple annotation error. Incorporating conflicting annotations without accounting for this structure can introduce systematic bias into downstream benchmarks and training datasets. MetaMP was therefore designed to make such cases explicit, enabling researchers to systematically distinguish well-supported annotations from those that require contextual interpretation or expert reassessment.

## Results

This section summarizes findings from the live MetaMP snapshot and the synchronized publication artifact. Results are organized into 7 areas: (a) integrated resource snapshot and source contributions, (b) discrepancy landscape and cross-source concordance on the expert benchmark, (c) transmembrane-segment benchmarking, (d) broad structural-group classification, (e) feature attribution and model interpretation, (f) representative interactive views, and (g) task-oriented user evaluation.

### Integrated resource snapshot and source contributions

At the live snapshot accessed on 2026 April 13, the harmonized MetaMP table contained 4,095 rows spanning 4,089 unique PDB codes. The linked source tables comprised 4,098 MPstruc rows spanning 4,092 (access date: 2026 March 27) unique PDB codes, 4,089 PDB rows, 2,612 OPM rows, and 3,976 UniProt rows. The harmonized release therefore retained near-complete MPstruc-PDB coverage while incorporating a specialized OPM membrane-orientation layer and a broader UniProt functional layer.

**Fig. 1.**
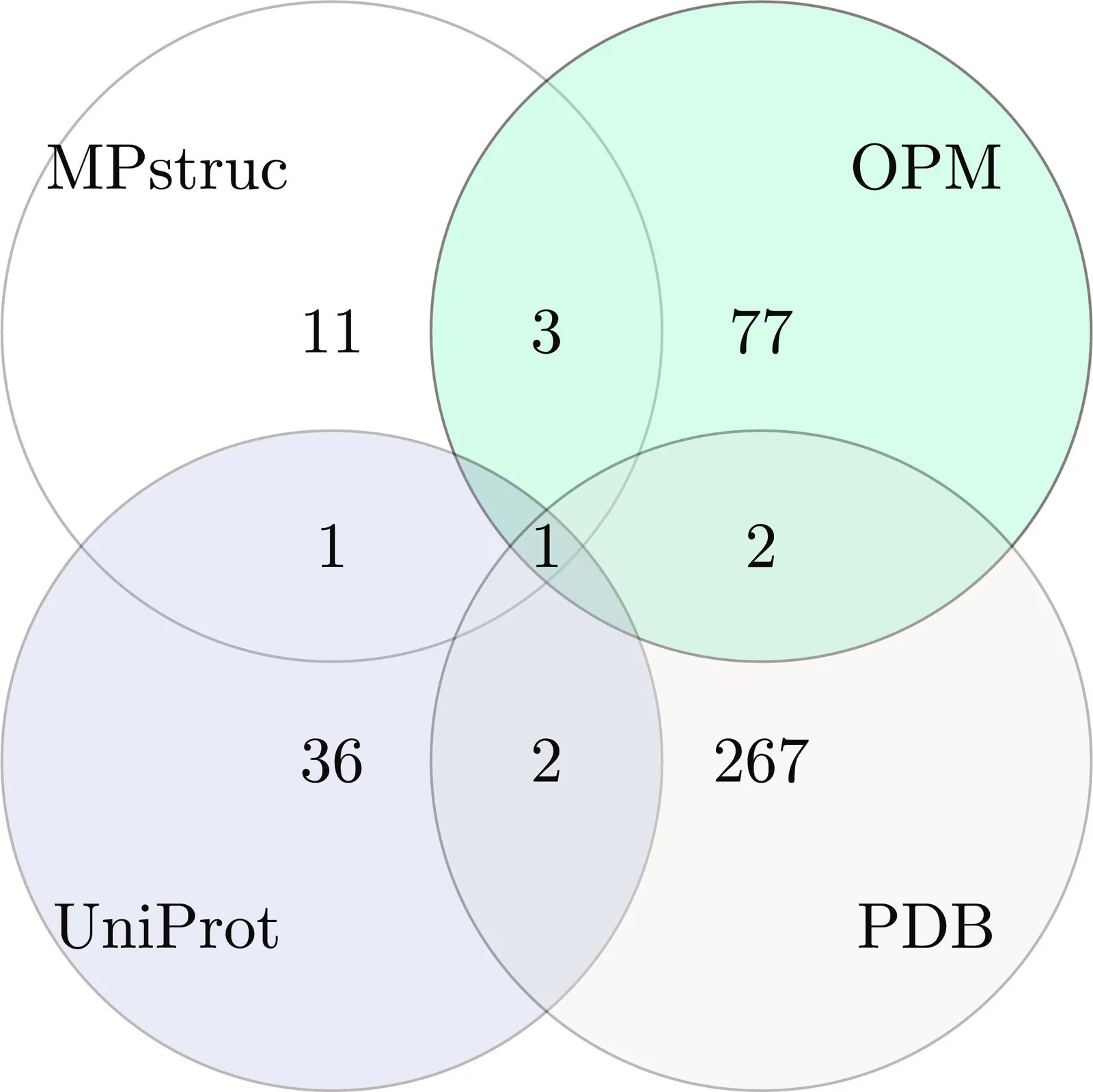
Venn diagram of the 4 dedicated protein databases integrated in MetaMP. The numbers inside each circle indicate the total attributes contributed by that source. The central value (1) represents the only attribute shared by all 4 sources: the PDB accession code (pdb_code). PDB has the most attributes (267), followed by OPM (77), UniProt (36), and MPstruc (11).

**Fig. 2.**
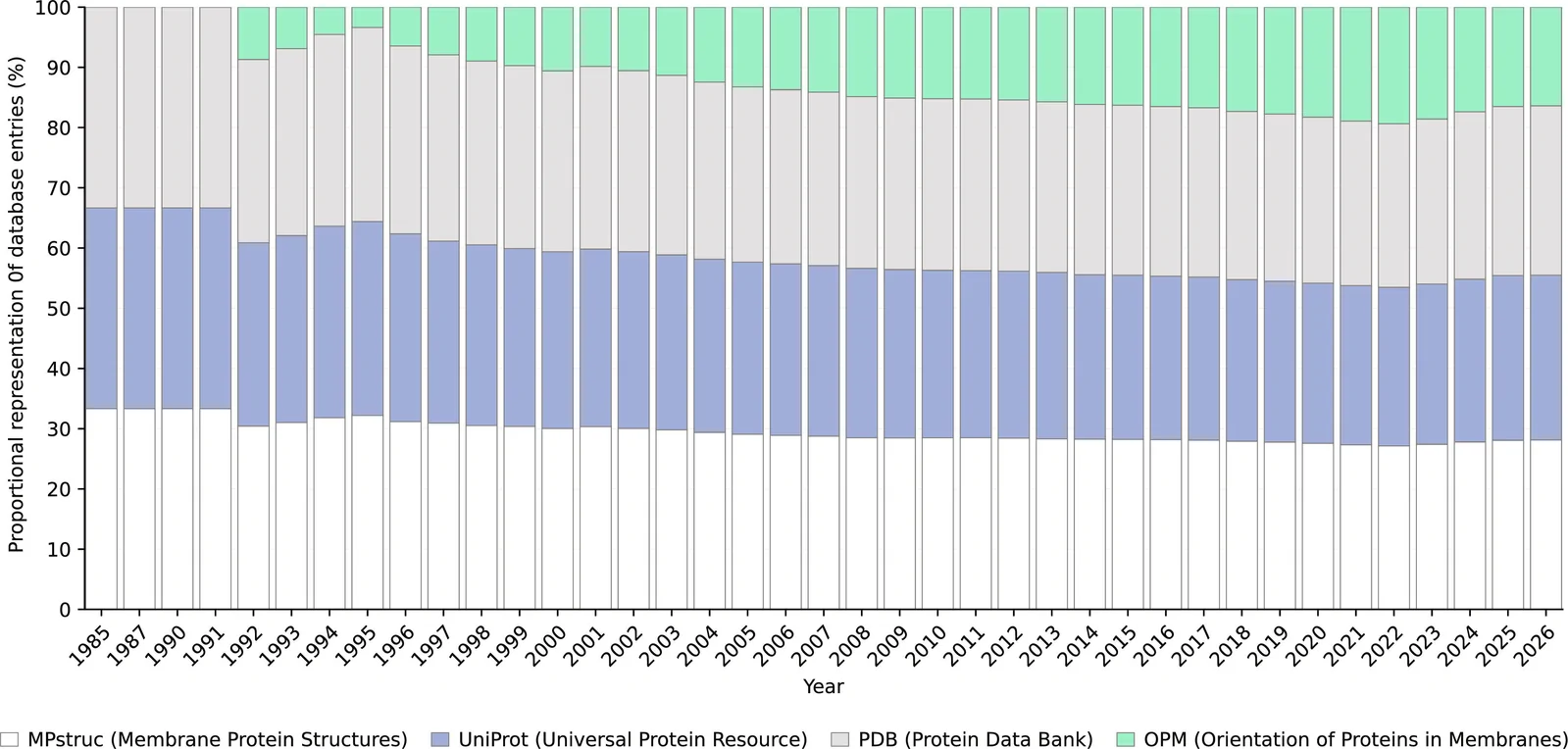
Comparative annual representation of MP entries from MPstruc, PDB, OPM, and UniProt. For each citation year, bars sum to 100% and show the cumulative proportional representation of the 4 source resources in the live MetaMP snapshot.

Table 1 summarizes source-level contributions to the live MetaMP release. The harmonized MetaMP table retained 282 annotation attributes in total, comprising 107 nominal and 175 quantitative fields. PDB contributed the broadest attribute layer (267 attributes), OPM contributed membrane-specific orientation descriptors (77 attributes), UniProt contributed 36 sequence-linked functional and taxonomic attributes, and MPstruc provided the broad-group and subgroup scaffold (11 attributes). Together, these layers yielded a unified but provenance-preserving MP resource anchored on 4,089 distinct structure observations. Expert broad-group labels were available for all 121 benchmark records, and explicit expert transmembrane-segment counts were available for 106 of them. The live snapshot excluded 3 unreleased PDB entries (9PJK, 7ROW, and 7UUV). Figure 1 shows the attribute overlap across the 4 source databases, and Fig. 2 shows the cumulative proportional representation of the 4 source resources over citation year. The structural coverage of the harmonized dataset reflects the historical adoption of 2 dominant experimental techniques. Cryo-electron microscopy has seen a marked rise in resolved structures alongside continued use of x-ray crystallography. For electron microscopy (EM)-annotated entries, mean reported resolution improved from 7.95 ± 2.47 Å in 2012 (*n* = 2) to 3.07 ± 0.48 Å in 2024 (*n* = 222), indicating a substantial gain in data quality over the past decade. X-ray diffraction entries show an overall mean reported resolution of 2.73 ± 0.65 Å (*n* = 1,763), consistent with its established precision for MP crystallography.

**Table 1.** Proportional contribution of each source database to MetaMP. The table reports membrane-protein observations and annotation attributes contributed by each source in the live application snapshot used for this manuscript.

Database	Observations ^a^	Source rows ^b^	Attributes ^c^	Nominal ^d^	Quantitative ^e^
MPstruc	4,092	4,098	11	11	0
PDB	4,089	4,089	267	94	173
OPM	2,612	2,612	77	50	27
UniProt	3,976	3,976	36	33	3
MetaMP	4,089	4,095	282	107	175

^a^ Observations: count of unique PDB accession codes present in each source.

^b^ Source rows: total number of retrieved records, which can exceed observations when repeated entries are retained for distinct states or conditions.

^c^ Attributes: total number of annotation columns available for each entry.

^d^ Nominal: categorical attributes with discrete labels.

^e^ Quantitative: numeric attributes amenable to arithmetic operations.

### Discrepancy landscape and cross-source concordance on the expert benchmark

Quality-control screening of the harmonized dataset identified 16 non-empty PDB Code Changed mappings linking obsolete accessions to validated replacement identifiers; representative examples include 5W7L→8G1N, 5G1J→7PDC, 3WXV→6KS0, and 3J8E→5TB0 (Table S1). These entries are framed as a curation and quality-control outcome of the harmonization process rather than as novel findings per se. Outlier detection also highlighted 6ZG5 as a notable entry: Its reported resolution of 40.0 Å reflects cryo-EM subtomogram averaging of a membrane-assembled complex rather than a data-entry error (Fig. S4).

Cross-source comparison of broad-group labels identified 121 entries (2.96% of 4,089 harmonized records) where MPstruc and OPM assigned conflicting classifications; these discrepancies are retained in the MetaMP reconciliation log rather than hidden by consensus-only integration. Not all identified disagreements represent annotation errors: Some reflect genuine biological complexity such as state-dependent membrane insertion or context-dependent topology, while others arise from differing curation conventions, database update timing, or taxonomic frameworks. The 121-record expert benchmark was assembled exclusively from this conflicting subset and is designed for evaluating annotation reconciliation under difficult conditions. It should not be interpreted as an estimate of disagreement prevalence across the full harmonized dataset.

Broad-group concordance was evaluated on the 121-record expert benchmark set at both strict and benchmark-aware levels (Table 2). Under strict label matching, OPM showed the highest agreement with the expert annotations, matching 96 of 121 records (79.34%), whereas MetaMP and MPstruc matched 25 of 121 (20.66%) and 17 of 121 (14.05%), respectively. The low strict MetaMP agreement should be interpreted in light of the training target: The classifier was trained to predict standardized MPstruc broad-group labels, whereas the held-out expert benchmark was intentionally assembled from entries where MPstruc and OPM disagreed. Thus, exact disagreement with expert labels partly reflects inherited MPstruc label conventions rather than an independently learned expert-annotation model. Benchmark-aware evaluation applies the live MetaMP collapsing rule, under which expert Bitopic labels are treated as compatible with predicted Bitopic, α-helical transmembrane, or β-barrel transmembrane labels. Under this biologically collapsed scheme, MetaMP achieved the highest agreement at 107 of 121 records (88.43%), followed by OPM at 98 of 121 (80.99%) and MPstruc at 95 of 121 (78.51%). Thus, the benchmark-aware scheme reversed the ordering observed under strict comparison: OPM led under canonical exact matching, whereas MetaMP showed the highest concordance under the broader biologically compatible evaluation. These concordance values should be distinguished from internal model-development metrics derived from separate training-workflow evaluations, because the 2 quantities address different evaluation questions.

**Table 2.** Cross-source broad-group concordance on the 121-row expert benchmark set. Strict agreement uses canonical standardized labels. Benchmark-aware agreement applies the live MetaMP collapsing rule in which expert Bitopic is counted as compatible with predicted Bitopic, α-helical transmembrane, or β-barrel transmembrane labels.

Source	Strict agreement	Benchmark-aware agreement
MetaMP	25/121 (20.66%)	107/121 (88.43%)
OPM	96/121 (79.34%)	98/121 (80.99%)
MPstruc	17/121 (14.05%)	95/121 (78.51%)

### Transmembrane-segment benchmarking

Transmembrane-segment benchmarking is reported here as an exploratory, entry-level characterization of agreement among heterogeneous topology evidence sources within the discrepancy-focused expert subset, rather than as a definitive predictor comparison. The benchmark was performed on 106 expert-curated records with explicit transmembrane-segment annotations, with predictor-specific overlap determined by the availability of non-null outputs (Table 3). Because the comparison includes sequence-based predictors, structure-derived membrane-plane methods, and annotation-derived segment counts that do not all operate on the same representation, findings should be interpreted in the context of this intentionally contested, nonrepresentative subset.

**Table 3.** Predictor agreement with expert transmembrane-segment counts on the 121-record discrepancy benchmark. Each predictor was evaluated on the subset of records with both an expert-assigned transmembrane-segment count and a non-null predictor output; this overlap is reported as *n*. Exact matches denote identical counts between a predictor and the expert annotation. MAE ± SD gives the mean absolute error and its standard deviation. Spearman ρ and Pearson *r* summarize rank-based and linear association with the expert count. Sequence-based predictors, structure-derived TMDET counts, and OPM-derived segment counts are included for comparison. This analysis is exploratory; the benchmark subset is limited to discrepancy-flagged records and is not representative of the full harmonized dataset. Boldface marks the highest exact-match rate within each of the TMAlphaFold and MetaMP predictor groups in this evaluation.

Predictor	*n*	Exact matches	MAE ± SD	Spearman *ρ*	Pearson *r*
**TMAlphaFold TMDET**	58	54/58 (93.1%)	2.29 ± 9.85	0.785	0.542
**MetaMP TMDET**	106	76/106 (71.7%)	3.95 ± 13.83	0.178	0.045
TMAlphaFold TOPCONS2	87	55/87 (63.22%)	4.01 ± 14.38	0.19	0.105
TMAlphaFold Phobius	87	55/87 (63.22%)	4.06 ± 14.44	0.098	0.066
TMAlphaFold HMMTOP	87	54/87 (62.07%)	4.06 ± 14.42	0.096	0.086
TMAlphaFold OCTOPUS	87	53/87 (60.92%)	4.06 ± 14.38	0.129	0.098
TMAlphaFold DeepTMHMM	106	64/106 (60.38%)	3.86 ± 13.4	0.226	0.323
TMAlphaFold MEMSAT	87	52/87 (59.77%)	4.11 ± 14.43	0.09	0.042
MetaMP TMHMM	106	63/106 (59.43%)	3.83 ± 13.11	0.149	0.311
MetaMP DeepTMHMM	106	61/106 (57.55%)	3.92 ± 13.14	0.22	0.267
TMAlphaFold Pro	87	50/87 (57.47%)	4.1 ± 14.42	0.137	0.067
MetaMP TMbed	106	60/106 (56.6%)	3.75 ± 12.98	0.05	0.343
TMAlphaFold SCAMPI-MSA	87	47/87 (54.02%)	4.11 ± 14.32	0.158	0.122
TMAlphaFold TMHMM	87	47/87 (54.02%)	4.15 ± 14.43	0.135	0.027
TMAlphaFold Prodiv	87	45/87 (51.72%)	4.32 ± 14.46	0.177	0.067
TMAlphaFold Philius	87	43/87 (49.43%)	4.18 ± 14.4	0.131	0.045
TMAlphaFold SCAMPI	87	40/87 (45.98%)	4.32 ± 14.3	0.13	0.038
OPM-derived segment count	95	19/95 (20%)	4.68 ± 10.44	0.276	0.634

Within this exploratory entry-level comparison, TMAlphaFold-linked TMDET achieved the highest exact-match rate, reproducing the expert count in 54 of 58 comparable records (93.10%), albeit on a reduced overlap. Among predictors evaluated on the full 106-record subset, MetaMP TMDET showed the highest exact-count agreement, matching the expert annotation in 76 of 106 records (71.70%), followed by TMAlphaFold-linked DeepTMHMM (64/106, 60.38%).

Within the TMAlphaFold-linked sequence-topology methods evaluated on the 87-record overlap, TOPCONS2 and Phobius shared the highest exact-match rate (55/87, 63.22%), followed closely by HMMTOP (54/87, 62.07%). Among the remaining 106-record predictors, MetaMP TMHMM matched the expert count in 63 of 106 records (59.43%), whereas MetaMP DeepTMHMM matched 61 of 106 (57.55%) and MetaMP TMbed matched 60 of 106 (56.60%). OPM-derived segment counts showed the lowest exact-match rate (19/95, 20.00%) but exhibited the strongest linear association with expert counts (Pearson *r* = 0.634), indicating that OPM-derived estimates captured overall segment magnitude despite reduced agreement at the exact-count level.

Overall, exact transmembrane-segment agreement varied substantially across evidence-source families and overlap subsets. These values describe agreement patterns within the current MetaMP export and should not be interpreted as a representation-invariant ranking of topology-predictor performance.

### Broad structural-group classification

The machine-learning workflow evaluated 36 model bundles, comprising 6 classifier families each trained under 2 modes (supervised and semi-supervised), across 3 representations of the same 7-feature OPM-derived input space: no dimensionality reduction (No-DR), principal components analysis (PCA), and uniform manifold approximation and projection (UMAP). After excluding the reserved discrepancy layer, the expert benchmark set, and the legacy manually curated benchmark layer, the exported training matrix contained 3,966 rows spanning 3,963 unique PDB codes. Within this matrix, 6 of the 7 OPM-derived descriptors (listed in the Supplementary Materials) were nonmissing for 2,500 rows, whereas topology_subunit was nonmissing for 2,445 rows, indicating that the workflow operated on a partially sparse but membrane-specific structural feature layer.

Performance was evaluated at 2 levels: internal model-registry performance and held-out expert-benchmark performance. Across all 36 bundles, the selected upload bundle (also the strongest semi-supervised bundle) was the No-DR Decision Tree model (Table 4 and Fig. S8). This model achieved an internal accuracy of 0.937 and weighted F1 of 0.928, together with a benchmark-aware (label-collapsed) expert accuracy of 0.884 and weighted F1 of 0.887. Under exact, noncollapsed label matching, performance decreased substantially (accuracy 0.207; weighted F1 0.142), reflecting the difficulty of strict label-space agreement across heterogeneous annotation schemes.

**Table 4.** Selected supervised and semi-supervised production bundles for broad structural-group classification. Internal metrics correspond to the stored model-registry internal evaluation for each bundle, whereas expert metrics were computed on the held-out 121-record expert benchmark under the benchmark-aware comparison scheme. Exact metrics use the noncollapsed label space.

Mode	Bundle	Internal acc.	Internal F1	Expert acc.	Expert F1	Exact acc.	Exact F1
Semi-supervised	No-DR Decision Tree	0.937	0.928	0.884	0.887	0.207	0.142
Supervised	UMAP Logistic Regression	0.883	0.833	0.884	0.885	0.124	0.073

The strongest supervised bundle in the current export was the UMAP Logistic Regression model, selected as the best supervised-mode bundle. It achieved internal accuracy of 0.883 and weighted F1 of 0.833, together with benchmark-aware expert accuracy of 0.884 and weighted F1 of 0.885. Under exact label matching, performance decreased further (accuracy 0.124; weighted F1 0.073). Thus, the best supervised bundle closely matched the selected semi-supervised bundle on benchmark-aware expert evaluation, but exhibited weaker internal-registry and exact-label performance.

The selected upload bundle was not the model with the highest internal weighted F1. The semi-supervised No-DR Gradient Boosting classifier achieved a slightly higher internal weighted F1 (0.934), but a lower expert-benchmark weighted F1 (0.877). This divergence between internal and held-out expert-benchmark performance reflects the MetaMP bundle-selection criterion, which prioritizes expert-benchmark behavior over internal registry metrics. Overall, internal validation and held-out expert-benchmark performance were not fully aligned: The strongest semi-supervised bundle emerged from the original feature space, whereas the strongest supervised bundle arose from the UMAP representation. The relative ranking of bundles, classifier families, and dimensionality-reduction modes is summarized in Figs. S9 and S10, and the exploratory PCA, t-distributed stochastic neighbor embedding (t-SNE), and UMAP projections used for visual inspection of the training matrix are shown in Fig. S11.

### Feature attribution and model-interpretation outputs

Explainability outputs were generated for the strongest interpretable tree-based production bundle, namely, the semi-supervised No-DR Decision Tree model selected for upload. Shapley additive explanations (SHAP) values were computed from a 500-row sample drawn from the eligible post-exclusion training matrix. Global mean absolute SHAP values are reported in Table S9. Membrane thickness ranked first (0.0881), followed by membrane topology out (0.0275) and subunit transmembrane segments (0.0133); the remaining 4 descriptors contributed substantially less (Fig. S7 and Table S9).

This ranking shows that the selected classifier relied primarily on membrane-thickness and topology-orientation information rather than on the more weakly contributing energetic or tilt descriptors. The class-specific beeswarm plots provide a more granular view of this dependency structure. Membrane thickness contributed strongly to the discrimination of both α-helical and β-barrel transmembrane proteins, whereas topology-related descriptors, particularly membrane-topology-in and membrane-topology-out, contributed more visibly to the separation of monotopic from transmembrane records. Overall, the SHAP analysis serves as an interpretability sanity check, confirming that the selected model relied on membrane-specific structural context rather than on arbitrary or uninformative features. These outputs should be interpreted as evidence of feature consistency within the model, not as independent biological discoveries about MP function.

### Eight interactive views

**Fig. 3.**
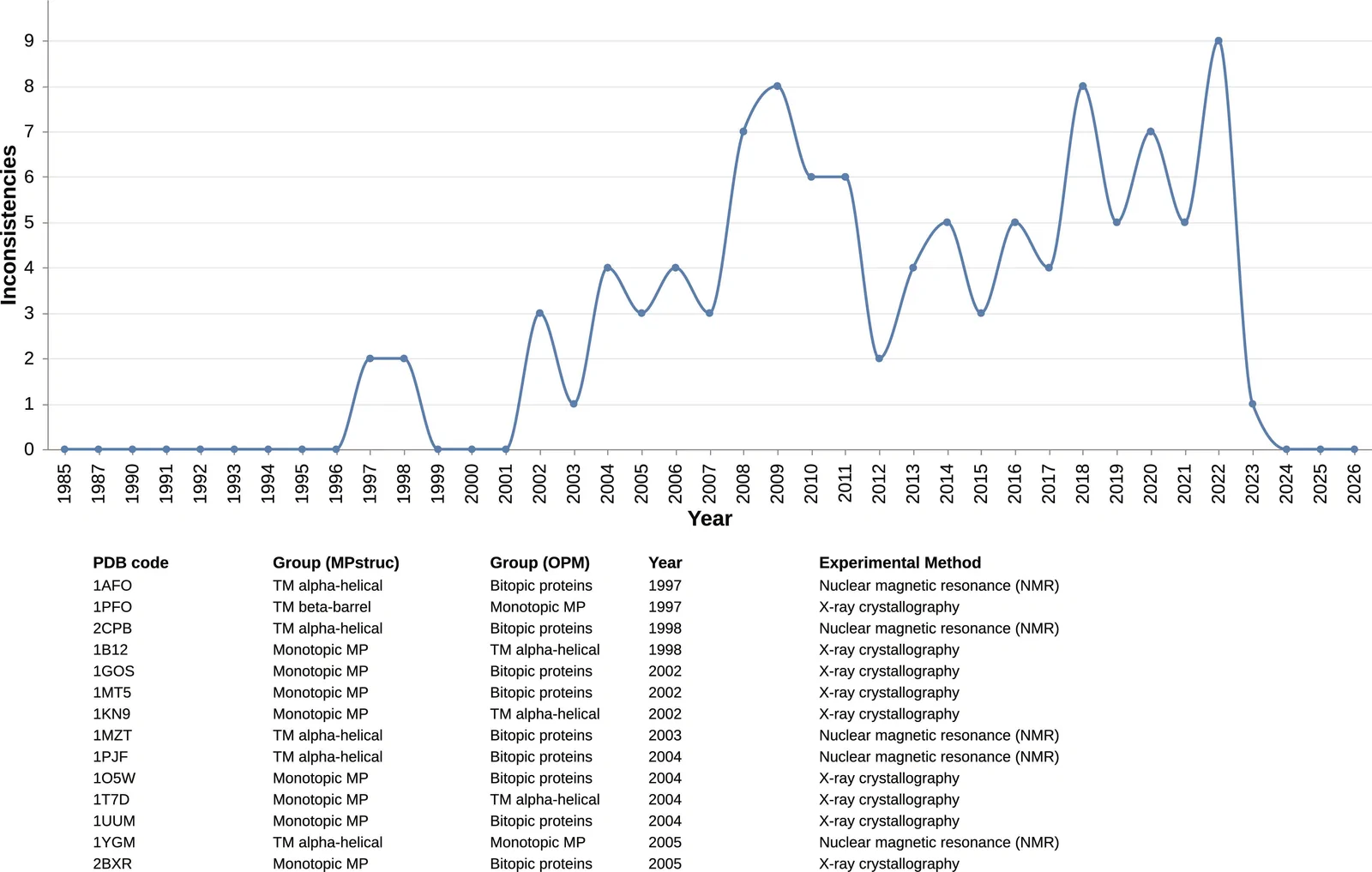
Data Discrepancy view for 1997–2005. The upper line chart shows classification inconsistencies between OPM and MPstruc over time. The linked table below lists the top 14 discrepant records, sorted by year, and supports brushing so that users can inspect the subset of records corresponding to selected regions of the chart. MP, membrane protein; TM, transmembrane; TM α-helical, transmembrane α-helical; TM β-barrel, transmembrane β-barrel.

The Data Discrepancy view (Fig. 3) visualizes MP classification inconsistencies across the 121 expert-reviewed records, with discrepancies concentrated between OPM and MPstruc. These cases were adjudicated by domain-expert review, examining source labels together with structural and topology evidence. Three representative entries illustrate distinct categories of reconciliation scenario.

1PFO (perfringolysin O, a cholesterol-dependent cytolysin) exemplifies a state-dependent discrepancy. The protein is secreted as a water-soluble monomer, binds cholesterol-containing membranes, and assembles into large oligomeric transmembrane pores [22]. MPstruc assigns it to a transmembrane group, reflecting this pore-forming state, while OPM classifies it based on a membrane-associated but not fully inserted structural state. The disagreement is biologically meaningful and cannot be resolved without specifying which functional state is under consideration.

1B12 (*Escherichia coli* signal peptidase I) illustrates a representation-level and curation-convention conflict. The full-length enzyme contains 2 N-terminal transmembrane segments and a periplasmic catalytic domain [23]; however, the deposited 1B12 structure represents the catalytic domain after removal of these N-terminal membrane segments. MPstruc records the entry as monotopic because the solved construct lacks the N-terminal helices and interacts with the membrane through a hydrophobic surface, whereas OPM retains a transmembrane/polytopic assignment based on the membrane-binding model of the full-length protein. The discrepancy therefore reflects whether the unit of classification is the deposited structural construct or the full-length biological protein, not a simple factual error.

1YGM (Mistic) is a noncanonical case. Mistic is a *Bacillus subtilis* membrane-integrating protein whose NMR structure helped explain its use as a fusion partner for improving heterologous MP expression [24]. Its database classification therefore reflects an unusual membrane-association and expression-helper context rather than a standard transmembrane-topology case. This entry illustrates benchmark records where disagreement arises from atypical biological or construct context rather than from a simple structural annotation error.

Together, these cases demonstrate MetaMP’s practical role as a reconciliation aid: The platform surfaces source-specific disagreement, preserves provenance, and links each ambiguity to the structural or biological context required for expert interpretation. They are included in MetaMP’s discrepancy export as documented reconciliation examples, not as claims of de novo biological discovery. The full set of 121 expert-curated records is provided in Table S8.

**Fig. 4.**
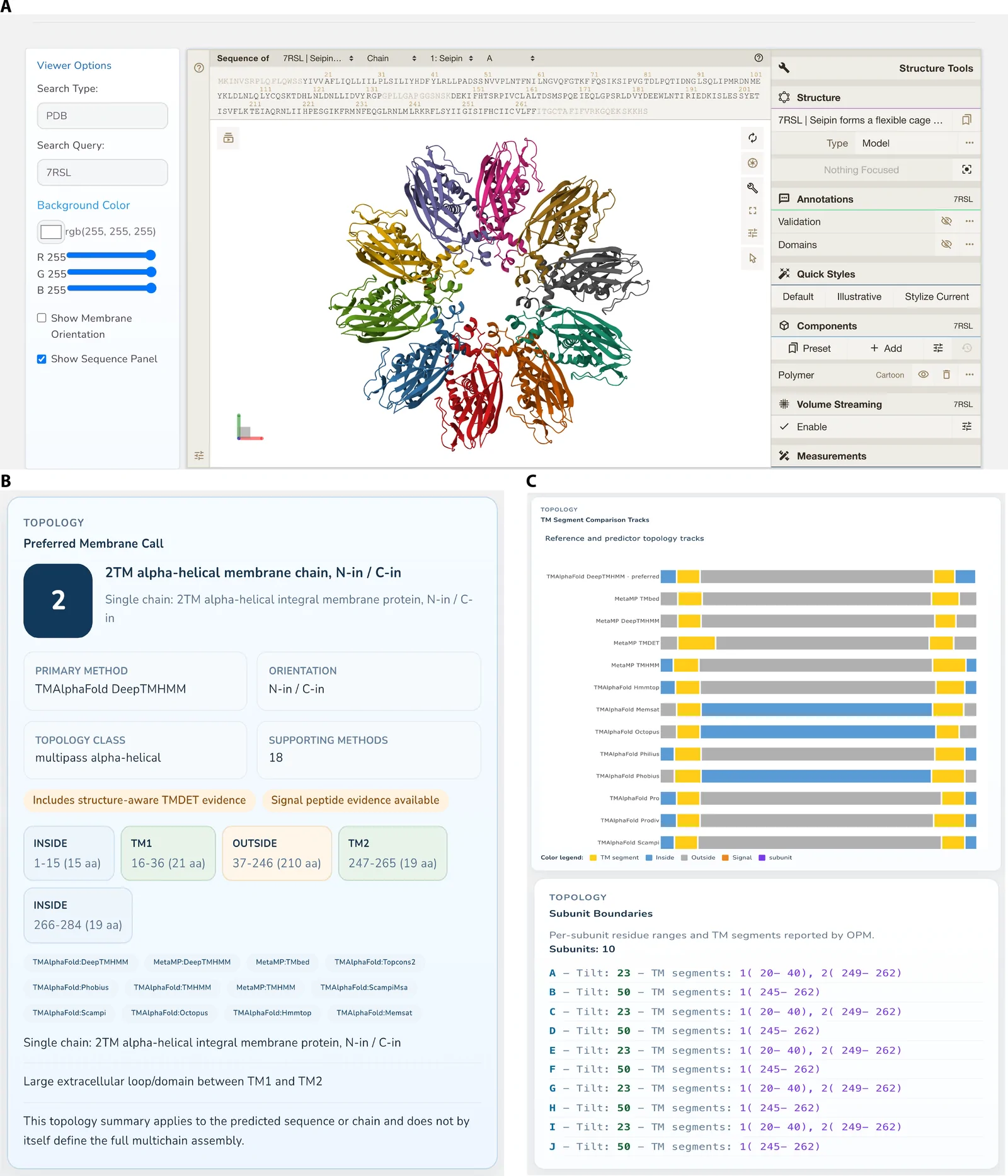
The Single-Entry Structural view. (A) Interactive 3D molecular viewer for PDB entry 7RSL (Seipin), showing the multi-subunit assembly with sequence and structure panels. 3D visualization is powered by Mol* [39]. (B) Topology summary panel reporting the preferred membrane call, transmembrane segment boundaries, orientation, and supporting predictor count for the selected chain. (C) The transmembrane segment comparison visualizes all normalized predictor outputs on a common residue axis using a shared color legend; OPM subunit annotations are located underneath.

The Single-Entry Structural view (Fig. 4) integrates protein structural, functional, and sequence information in a single panel. The center panel displays a 3-dimensional (3D) ribbon diagram; summary tables enumerate annotated structural features and functional annotations; and additional panels cover taxonomy, sequence characteristics, topology, and curated biological annotations.

### Task-oriented user evaluation

We conducted a task-oriented user evaluation to assess the usability of selected MetaMP views and users’ ability to complete representative analytical tasks efficiently and accurately. This evaluation assessed selected workflows rather than the full platform; findings should be interpreted as targeted evidence for task support in 2 representative views (Summary Statistics and Outlier Detection), not as a complete usability validation of all MetaMP functions. A total of 24 participants completed the evaluation. The sample comprised 13 male, 10 female, and 1 gender-undisclosed participant, and sociodemographic characteristics and domain expertise are reported in the Supplementary Materials (Table S3). All participants were volunteers and received no compensation, and the study was conducted in accordance with the ethical guidelines of the Centre for Artificial Intelligence at the Robert Koch Institute, Berlin, Germany.

Across the 3 tasks, the mean performance score (defined as the count of correct responses across all 6 task questions, scored on a 0 to 6 scale) was 4.21 ± 0.98 [95% confidence interval (CI) 3.80 to 4.62], and the combined training and testing phases required 9.35 min on average (95% CI 5.70 to 12.99). Task timing values were verified as non-negative across all participants and tasks; no anomalous values were identified in the raw records. The training phase averaged 1.90 min per task (95% CI 1.16 to 2.64) and the testing phase averaged 1.22 min per task (95% CI 0.63 to 1.80), indicating that participants completed the testing phase 35.97% faster than training, consistent with a short learning effect after initial interface exposure. Mean total completion times per task were 3.13 min for Task 1 (95% CI 1.74 to 4.53), 2.14 min for Task 2 (95% CI 0.92 to 3.36), and 4.08 min for Task 3 (95% CI 2.40 to 5.76), confirming Task 2 as the least demanding and Task 3 as the most demanding. Full descriptive user-study summaries, including participant-level means, standard deviations, and 95% CIs for all key measures, are provided in Table S7.

Three hypotheses guided quantitative analysis. H1 (null: no difference in completion time between training and testing phases) was tested using a Wilcoxon signed-rank test on paired training-versus-testing completion times, which yielded W=46.0 (P=0.0020, r=0.61, large effect), supporting rejection of H1 and confirming that structured training improved task efficiency. H2 (null: no difference in completion time across tasks) was examined using a Friedman test, which returned $\chi^{2}=8.58$ (P=0.0137, Kendall’s W=0.18, small-to-medium effect), and a repeated-measures analysis of variance (ANOVA) sensitivity analysis (F=4.39, P=0.0180), both confirming that completion time differed significantly across tasks. H3 (null: no association between completion time and response correctness) was assessed using a logistic regression with completion time as the independent variable, which estimated a small negative coefficient (coefficient =−0.1555, P=0.0504; pseudo-$R^{2}=0.0221$; Pearson r=−0.170), consistent with the clustered generalized estimating equations (GEE) sensitivity estimate (coefficient =−0.1426, P=0.0126). This provides only limited evidence for a meaningful speed–accuracy trade-off; the H3 analysis should be considered exploratory because the training phase included guidance by design.

Usability was assessed using an adapted 14-item questionnaire, of which items 5 to 14 formed a 10-item system usability scale (SUS)-style block scored on the standard 0 to 100 scale. After exclusion of incomplete SUS responses, the mean usability score was 72.81 (median 76.25, SD 15.77; 95% CI 66.16 to 79.47). A score above 68 is commonly interpreted as above average on the standard SUS scale; the point estimate therefore lies in the above-average to Good range [25], not at the excellent end of the scale. Because the 95% CI slightly overlaps the threshold of 68, the data support an above-average point estimate but not a stronger claim that the population mean clearly exceeds that benchmark. Most participants rated the system in the acceptable-to-good range, although the distribution showed noticeable variability across users (Fig. S5). Free-text comments most frequently highlighted system speed and reliability as strengths, while constructive criticism identified issues in chart positioning, drop-down behavior, and interface responsiveness, which informed iterative refinements to the platform.

## Methodology

MetaMP was designed as an integrated analytical platform for metadata-driven MP reconciliation and analysis, and the methodology accordingly spans data harmonization, discrepancy detection and reconciliation support, supervised and semi-supervised machine-learning components, topology benchmarking, and user-facing analytical workflows evaluated through task-based studies. These components are treated as interacting subsystems of a single platform rather than as independent contributions.

### Data sources and system design

MetaMP harmonizes MP annotations from 4 complementary resources: MPstruc [26], RCSB PDB [27], OPM [28], and UniProt [29]. These were selected because they capture complementary aspects of MPs with partial redundancy. MPstruc provides curated broad structural groups and subgroups; PDB provides structure-level, experimental, and bibliographic metadata; OPM provides membrane-orientation annotations, including spatial orientation within lipid bilayers, membrane thickness, tilt angle, and Gibbs free energy of transfer; and UniProt provides sequence-linked, taxonomic, and functional metadata, including molecular functions, cellular components, biological processes, and protein–protein interaction information. The purpose of integrating these sources was not to replace their original curation, but to make their overlaps, gaps, and disagreements explicit within a single analytical framework. MetaMP was designed to operate primarily at the annotation and metadata layer so that integration, discrepancy analysis, and downstream model-assisted classification remained interpretable within a provenance-controlled framework.

MPstruc organizes MPs at 3 hierarchical levels: groups, subgroups, and individual entries [26,30,31]. At the group level, proteins are categorized by their mode of membrane interaction; monotopic proteins contact only one leaflet of the bilayer, while transmembrane proteins span it via α-helices or β-barrels. MPstruc served as the initial anchor for data collection because of its human-curated nature, which mitigates problems arising from fully automated annotation, although curation error remains a recognized limitation [30,31]. PDB accession codes and UniProt accessions were used as the principal identifiers to retrieve and link records across all 4 sources.

MetaMP’s integration scope is intentionally bounded. PDBTM and UniTmp were considered but not included at this stage: PDBTM’s emphasis on transmembrane topology overlaps substantially with OPM’s orientation-based annotations, while UniTmp integrates existing annotation layers rather than supplying independent primary metadata. Including these sources is a planned extension. In the live snapshot analyzed here, the source tables contained 4,092 MPstruc MP entries (4,098 source rows), 4,089 PDB MP records, 2,612 OPM MP records, and 3,976 UniProt MP records. After harmonization, the MetaMP table contained 4,089 entries corresponding to distinct PDB codes. These counts reflect MPstruc’s curated scope and the overlaps and gaps across PDB, OPM, and UniProt, rather than a claim to exhaustive coverage of all MP structures in the PDB.

MetaMP follows a 3-layer architecture comprising a data layer, an application layer, and a presentation layer [32]. This separation keeps ingestion, harmonization, computation, and user interaction modular. The platform is implemented as a web-based system with a relational database backend and containerized deployment (Supplementary Materials). Data export in the current production workflow is supported in CSV, JSON, PNG, PDF, and selected HTML outputs, enabling integration with external bioinformatics tools and publication workflows. Full implementation details, including the software stack and deployment configuration, are available in the public source repository (see Data Availability).

### Data layer: Extraction, harmonization, and provenance

The data layer was implemented as an extract-transform-load (ETL) workflow [33], as shown in Fig. S2. Source-specific artifacts were collected into a staging area for temporary processing before being loaded into the MetaMP database, separating source parsing from application logic and maintaining data integrity during transfer. The harmonization workflow comprised 6 operations:

1. Data cleansing removed malformed characters, redundant whitespace, and formatting inconsistencies. For example, organism labels appearing as “E. Colli”, “E. Coli”, or “Escherichia Coli” were standardized to a single canonical form.
2. Filtering restricted extraction to relevant records. Only PDB records corresponding to MP structures listed in MPstruc were retained.
3. Normalization standardized semantically equivalent values across sources, including broad-group labels and selected metadata fields.
4. Verification confirmed that staged artifacts were transferred and loaded correctly into intermediate tables within the MetaMP database. In practice, this included row-count consistency checks, schema and column-alignment checks, and identifier-integrity checks across staging and target tables.
5. Restructuring converted compound fields into machine-readable columns. For example, “exptl_crystal_grow” was split into derived columns prefixed with the parent field name.
6. Lookup-based merging linked records across resources using PDB codes and UniProt accessions.

After lookup-based merging, harmonized records were collapsed to one row per distinct PDB accession for downstream application and analysis workflows while preserving source-specific fields and replacement mappings within the integrated record.

A key design decision was that harmonization preserved source-specific values rather than overwriting them. Conflicting labels remained attached to the harmonized record so that disagreement stayed visible and traceable, supporting reconciliation rather than silent aggregation. Legacy and replacement PDB mappings were also retained so that obsolete entries could be linked to current identifiers without losing historical context.

The ingestion workflow was incremental where possible. Existing records were updated by key, and non-empty live values were not overwritten by empty incoming fields during ordinary refreshes, preserving derived annotations such as recovered sequences, review state, topology-prediction outputs, and model-derived labels across repeated updates. Recovery mechanisms resume operations from the point of interruption in the event of a load failure.

### Application layer: Retrieval, reconciliation, and updating

The application layer exposes the harmonized dataset via a RESTful API and provides search, filtering, discrepancy review, and model-assisted annotation workflows while remaining decoupled from data-ingestion and update processes.

Search and retrieval operated at both record and field level. Records could be queried by identifiers, names, and annotation fields and filtered by structural class, topology, organism, resolution, and related metadata. For discrepancy review, the platform supported server-side pagination, search, and filtered export so that large candidate sets remained inspectable in production.

MetaMP stored source-specific annotations, identified discrepancies, and exposed discrepant records for review rather than forcing a hidden consensus. Replacement mappings, provenance, review state, and source-specific summaries were recorded so that discrepancies remained interpretable and auditable.

Continuous updating was managed through background jobs that refreshed datasets, derived artifacts, machine-learning outputs, and validation reports at regular intervals. These jobs are executed as Celery tasks with periodic maintenance scheduling coordinated via Celery beat; the currently configured built-in periodic schedule includes monthly production maintenance. Long-running topology-prediction workflows store completed batches incrementally, allowing interrupted runs to resume from the last successfully processed batch.

### Expert reference set, discrepancy assembly, and concordance

To support reconciliation and held-out evaluation, an expert benchmark set of 121 MP records spanning publication years 1997–2023 was assembled. Of the 4,089 distinct PDB codes in the harmonized membrane_proteins table, cross-source label comparison between MPstruc and OPM identified 121 entries where the 2 databases assigned conflicting broad-group classifications to the same structure. An entry was flagged when one source classified a protein as monotopic and the other as transmembrane, or when the 2 differed in transmembrane subtype. All flagged entries were retained; no additional filtering was applied.

Flagged candidates were examined individually by a domain expert, who reviewed source labels, structural and topology evidence, and published descriptions before assigning a confirmed broad-group label. Expert broad-group labels were assigned for all 121 records. Transmembrane-segment counts were additionally recorded for 106 entries; the remaining 15 were classified as monotopic and assigned no transmembrane-segment count, as monotopic proteins do not traverse the bilayer. This benchmark is not a universal gold standard: Its scope is limited to entries where cross-source disagreement existed at database assembly time, and it should be interpreted as targeted evidence for disagreement handling rather than as an estimate of global annotation quality.

MetaMP assembled discrepancy records from both static and live sources. The expert benchmark contributed broad-group labels and transmembrane counts alongside the original OPM and MPstruc source labels. Model-based predictions were taken from live production artifacts; transmembrane counts and predicted regions were assembled from the live predictor store and discrepancy export workflow. Expert annotations were kept fixed for comparison, while predicted labels and topology outputs remained dynamic. All labels were standardized before comparison to avoid artificial discrepancies arising from spelling or casing differences.

Class-based concordance was evaluated at 2 levels. Strict agreement was computed after standardizing source labels to canonical broad-group forms. Benchmark-aware agreement applied a biological collapsing rule whereby expert Bitopic labels were treated as compatible with predicted Bitopic, Transmembrane proteins: α-helical, or Transmembrane proteins: β-barrel, while expert Monotopic remained strictly monotopic. OPM and MPstruc labels were compared to expert annotations directly; MetaMP model concordance was computed from the held-out prediction file of the currently selected model bundle.

For concordance analysis, source labels were normalized to canonical broad-group categories before comparison. These categories followed the definitions introduced above: monotopic, bitopic, and transmembrane. Original MPstruc, OPM, PDB, and UniProt annotations were retained alongside the harmonized labels so that source-specific terminology remained inspectable.

A discrepancy was defined as a mismatch in broad structural-group assignment, transmembrane-segment count, or transmembrane-boundary support across one or more sources (see Discrepancy and scientific-assessment flags). Discrepant entries were surfaced in the discrepancy interface for review. This workflow was assistive rather than automated: MetaMP preserves provenance, surfaces disagreement, and records curator review outcomes where available, but does not resolve disagreements automatically.

### Expert curation and validation

All benchmark labels were assigned by a domain expert with experience in MP structure determination and topology annotation. For each flagged entry, the expert reviewed the original MPstruc and OPM annotations, associated PDB and UniProt records, and primary publications describing the structure and its membrane context.

Broad structural-group labels (monotopic, bitopic, and transmembrane subtypes) were assigned using a consistent decision protocol that considered the reported membrane association, oligomeric state, and experimentally supported transmembrane segments. When necessary, additional structural evidence such as orientation in OPM, TMDET membrane-plane placement, and visual inspection of the 3D structure were consulted to resolve ambiguous cases.

Transmembrane-segment counts were recorded only when a clear consensus could be established from experimental and structural evidence. Entries for which the expert judged the available information insufficient for a reliable segment count were retained in the benchmark for broad-group comparison but excluded from transmembrane-count aggregation. All expert decisions were made independently of MetaMP model predictions, which were only compared against the final expert labels.

The expert-assigned labels were not cross-checked by an independent second annotator. This single-annotator design reflects a practical constraint and introduces a risk of individual-specific bias, particularly in ambiguous cases such as state-dependent proteins or noncanonical membrane-associated constructs. Users should treat these labels as expert-informed reference assignments rather than as formally adjudicated gold standards. Entries flagged as context-dependent or noncanonical in MetaMP’s scientific-assessment layer carry the highest residual uncertainty and would benefit most from future multi-expert review.

### Transmembrane-segment benchmarking

Expert annotations and experimental observations serve as the reference standard against which topology predictors are evaluated. Predictors are not used to validate experimental records; disagreement between a predictor and an expert annotation flags the entry for closer inspection, following standard practice in topology-predictor benchmarking [21,34].

MetaMP integrates a TMAlphaFold-linked predictor layer [21] that contributes per-method transmembrane-segment summaries for DeepTMHMM, HMMTOP, MEMSAT, OCTOPUS, Philius, Phobius, PRO, PRODIV, SCAMPI, SCAMPI-MSA, TMHMM, and TOPCONS2, together with auxiliary SignalP summaries and the structure-based TMDET membrane-plane output where available. TMAlphaFold-linked methods are ingested through the TMAlphaFold synchronization workflow rather than run independently. Because the TMAlphaFold API imposes strict usage limits, restricting comprehensive batch analysis, MetaMP incorporates a local fallback pipeline that is invoked automatically when the TMAlphaFold synchronization returns no prediction for a given entry or when the associated UniProt accession is absent from the TMAlphaFold database. Four predictors were selected for the fallback pipeline on the basis of open availability and licensing: TMbed for sequence-topology prediction [34], TMHMM and DeepTMHMM as established sequence-based baselines, and TMDET for structure-based membrane-plane estimation. OPM-derived transmembrane-segment counts were retained as a comparative baseline.

Expert transmembrane counts were parsed using a normalization routine that extracts the leading integer from strings such as 1**, 1 *, 1x8, or 0**, evaluating these as 1, 1, 1, and 0, respectively. Blank expert fields are excluded from aggregation; predictor-specific benchmark sizes therefore depend on the overlap between nonmissing expert and predictor counts. Agreement was summarized using exact-match rate, mean absolute error, standard deviation of absolute error, Spearman’s ρ, and Pearson’s r.

MetaMP integrates annotations at the PDB-entry level rather than at individual chain resolution. For oligomeric assemblies, transmembrane-segment counts refer to the deposited entry as a whole. Because many predictors operate on sequence or chain representations, whereas MetaMP reconciles annotations at PDB-entry level, these comparisons are interpreted as approximate and are qualified by contextual flags rather than treated as exact chain-level equivalences. The Multichain Context caution flag identifies such entries as unsuitable for straightforward single-chain topology comparison.

### Machine-learning module for broad structural-group classification

To support broad structural-group assignment, we implemented supervised and semi-supervised machine-learning workflows for 3 categories: monotopic, α-helical transmembrane, and β-barrel transmembrane. The goal was to provide a reproducible prediction baseline, not to replace expert classification. The classifier operates only on harmonized metadata and database annotations integrated from MetaMP’s 4 source resources (MPstruc, RCSB PDB, OPM, and UniProt); it does not use primary amino-acid sequence, atomic-coordinate features, multiple-sequence alignments, or learned protein-language-model embeddings.

Target variable. The target variable was the standardized MPstruc broad-group label, normalized before fitting so that semantically identical categories were not treated as separate classes.

Data preparation. Training data excluded 3 reserved benchmark layers: the live discrepancy-review export, the expert benchmark set, and a legacy manually curated list. In the production snapshot analyzed here, the union of these reserved layers comprised 126 PDB codes, yielding a training scope of 3,966 rows spanning 3,963 unique PDB codes. Preprocessing included broad-group normalization, retention of required machine-learning feature columns even when sparse, in-pipeline imputation of missing numeric values, encoding of categorical topology fields, and exclusion of rows lacking the target broad-group label.

Feature selection. Seven OPM-derived metadata descriptors were used and held fixed for the production workflow. Four were treated as numeric variables: thickness, subunit_segments, tilt, and gibbs. Three were treated as categorical topology descriptors: topology_subunit, membrane_topology_in, and membrane_topology_out. The topology descriptors were encoded inside the training pipeline using label maps fit on the retained post-exclusion training scope. This feature set was informed by prior exploratory screening and then fixed for the production workflow. Feature selection was intentionally restricted to OPM-derived metadata descriptors because MetaMP operates at the annotation and metadata layer rather than as a sequence- or structure-based topology predictor. This restriction isolates membrane-context metadata as an independent signal for broad structural-group classification, avoids confounding from sequence-derived features already embedded in several topology tools, and preserves interpretability within the same provenance-controlled annotation layer used for discrepancy analysis. Feature interpretability was assessed using SHAP [35].

Semi-supervised learning setup. Semi-supervised models used a labeled training subset together with a withheld-label subset derived from the same retained labeled scope by masking labels. The withheld-label subset was not an external unlabeled corpus; it was created from the retained labeled dataset to evaluate self-training within a controlled internal setting. The production workflow first stratified the labeled base matrix into a labeled subset (70%) and a withheld-label subset (30%) using random_state = 42. Within the labeled subset, the semi-supervised wrapper further stratified data into 80% train and 20% test splits. Missing values in the assembled input matrix were median-imputed, and features were scaled using StandardScaler. The withheld-label subset was transformed with the same fitted imputer and scaler, concatenated with the labeled training subset, and assigned a label −1 for self-training. Each base classifier was wrapped with scikit-learn 1.3.2’s SelfTrainingClassifier using default parameters: criterion = “threshold”, threshold = 0.75, and max_iter = 10. A fixed conservative threshold of 0.75 was selected to prioritize pseudo-label precision over pseudo-label volume under the default self-training framework. Training terminated when the iteration limit was reached, no new pseudo-labels met the threshold, or all withheld-label samples had been labeled. All 6 semi-supervised classifier families used identical self-training wrapper settings. Semi-supervised bundles were evaluated using the labeled hold-out test split and the same 121-entry expert benchmark set; pseudo-labeled samples were excluded from expert-benchmark evaluation and discrepancy exports. For semi-supervised bundles, registry fields named cv_* store this internal labeled hold-out performance rather than fold-wise cross-validation. In both supervised and semi-supervised settings, all random-number generators were initialized with random_state = 42.

For model-bundle training and benchmarking, MetaMP evaluated 3 production views of the same feature set: the original feature matrix (No-DR), a 2-component PCA view, and a 2-component UMAP view. Exploratory PCA, t-SNE, and UMAP projections were also generated separately for visual inspection of the training records, but t-SNE was not used as a production model-bundle transform because it is comparatively sensitive to sample size and duplicated points and does not provide the same straightforward reusable transform path for production bundle export.

Evaluation and bundle selection. Performance for supervised bundles was assessed by 5-fold cross-validation on the supervised training partition and by held-out expert benchmark evaluation; for semi-supervised bundles, it was assessed by labeled hold-out evaluation and held-out expert benchmark evaluation. Accuracy, precision, recall, and weighted F1 were computed at both internal-evaluation and expert-benchmark levels. The production bundle was selected by ranking candidates on benchmark-aware held-out weighted F1, then benchmark-aware held-out accuracy, then the internal weighted F1 stored in cv_mean_f1 (5-fold cross-validation for supervised bundles and labeled hold-out evaluation for semi-supervised bundles), with dimensionality-reduction choice used as a later tie-breaker.

Explainability. SHAP outputs [35] were generated for the strongest interpretable tree-based production bundle, restricted to original-feature (No-DR) bundles so that explanations remained tied to the underlying OPM-derived descriptors rather than to reduced latent coordinates. A global bar plot of mean absolute SHAP values and class-specific beeswarm plots were produced from a 500-row random sample drawn from the eligible training matrix after benchmark exclusions, using the same random seed of 42 for subsampling.

### Discrepancy and scientific-assessment flags

MetaMP computes 3 layers of record-level interpretability signals: discrepancy flags from cross-source comparison of annotations and topology predictions; scientific-assessment flags from curated overrides, keyword rules, structure context, and replacement metadata; and benchmark-decision fields that classify each record as excluded, included with caution, or high-confidence.

Discrepancy flags: Three discrepancy indicators are computed per record.

1. Group disagreement. Raised when broad-group labels from expert annotation, OPM, MPstruc, and machine-learning prediction do not resolve to a single standardized category.
2. TM count disagreement. Raised when transmembrane-segment counts differ across expert annotation and predictor or reference sources.
3. TM boundary disagreement. Evaluated only when at least one OPM boundary annotation and one comparable predictor annotation are available and both sources report the same segment count. Membrane regions are converted to ordered (start, end) boundary signatures; disagreement is raised when any paired segment differs by strictly more than 5 residues at either coordinate. A tolerance of 5 residues was chosen to reduce artificial disagreement caused by minor annotation-boundary offsets between sources while still flagging materially different membrane-segment placements. Comparable predictor sources are TMbed, DeepTMHMM, TMHMM, Phobius, TOPCONS, CCTOP, and TMDET.

Scientific-assessment flags: MetaMP computes a set of scientific caution indicators to identify records that may be unsuitable for straightforward sequence-based topology benchmarking under the operational rules defined below. These indicators are heuristic rather than definitive.

The top-level indicator, benchmark recommended, is true unless the record is flagged as context-dependent, noncanonical, or obsolete/replaced or an explicit override marks it as unsuitable. Explicit overrides take precedence over keyword-based evidence; replacement and obsolescence are always exclusion criteria. Four flags are exposed in the application:

1. Context-dependent topology. Raised when topology may depend on biological state or soluble-to-membrane transition rather than sequence alone. Sources include entry-level overrides and keyword rules matching pore-forming toxins, hemolysins, cytolysins, prepore or assembly-dependent language, conformational switching, amphitropic association, and related terminology. Derived internally from state_dependent and soluble_to_membrane_transition.
2. Noncanonical membrane case. Raised when a record is unlikely to be a standard benchmarking target. Sources include overrides and keyword rules matching fusion partner, expression helper, Mistic, carrier protein, and related construct descriptions.
3. Multichain context. Raised when the deposited structure contains more than one chain, indicating that per-entry topology interpretation may require chain-level or assembly-level inspection.
4. Obsolete or replaced. Raised when PDB replacement metadata indicates that the entry is obsolete or superseded, causing exclusion from the recommended benchmark subset.

MetaMP also stores supporting metadata: a categorical confidence score (none, low, medium, high) from the strongest matched rule; matched_rule_ids identifying which rules fired; soft_review_reasons for lower-confidence signals that recommend inspection without triggering exclusion; context_reasons recording the specific causes of context dependence; free-text notes explaining each assignment; a boolean review_recommended field raised when softer signals or multichain ambiguity warrants manual inspection; and a benchmark_exclusion_reasons list recording all exclusionary conditions applied to the record.

Benchmark-decision layer: Scientific-assessment outputs, discrepancy status, and review metadata are combined to derive include_in_benchmark, high_confidence_subset, benchmark_status, and a human-readable benchmark_reason. A record is marked include_in_benchmark when it has at least one inclusion reason, currently either observed disagreement or a discrepancy-review status of accepted, reviewed, or uncertain, provided that the review status is not rejected and that the record does not lack both the expert broad-group label and the expert transmembrane-segment count. The stricter high_confidence_subset field is true only when the record is not rejected or replaced, carries no context-dependent, noncanonical, or multichain flag, has no transmembrane-boundary disagreement, shows agreement between expert and predicted broad-group labels or explicit expert confirmation of the predicted group, and, when an expert transmembrane-segment count is available, shows agreement between the expert count and the available TMbed, DeepTMHMM, and TMDET counts. These rules yield 4 benchmark-status levels: high_confidence_subset for records passing the stricter subset criteria, included_with_caution for records retained in the broader benchmark despite caution conditions, not_recommended for records considered unsuitable for straightforward sequence-only topology benchmarking because of scientific-assessment exclusions, and excluded for records that do not satisfy the inclusion criteria or have review status rejected. MetaMP also stores explicit inclusion_reasons and exclusion_reasons to keep benchmark decisions auditable.

Rule sources and transparency: All indicators are generated by explicit rule-based logic in publicly inspectable MetaMP source modules, drawing on 4 evidence sources: entry-specific curated overrides; keyword regular-expression rules applied to record names and descriptions; structure-context summaries such as chain count; and PDB replacement metadata. These indicators were designed for interpretability and auditability rather than statistical optimization and should be read as transparent scientific heuristics, not as a trained classifier.

### Presentation layer and interactive views

The presentation layer operationalizes the underlying data integration, discrepancy, topology, and machine-learning components through a set of coordinated interactive views. Rather than being contributions in their own right, these views provide the user-facing mechanism through which provenance-preserving integration, disagreement-aware reconciliation, topology comparison, model-assisted annotation, and expert review are examined and acted upon.

High-level views (Homepage, Overview, Summary Statistics, Database, Exploration, and AI Use Cases) provide descriptive statistics, tabular access, filtering, and faceted exploration of the harmonized dataset and derived model outputs. They support tasks such as examining database composition over time, inspecting distributions of experimental methods and resolutions, and exporting filtered subsets for external analysis.

Several views are more directly tied to the core methodological contributions:

1. Data Discrepancy. This view operationalizes the disagreement-aware reconciliation framework. Two coordinated panels surface records where MPstruc, OPM, expert labels, and model predictions disagree in broad structural-group assignment, transmembrane-segment count, or boundary support. The upper panel combines a line chart of annual inconsistency counts with a scrollable table listing PDB code, conflicting group assignments across OPM, MPstruc, and predictor outputs, expert labels, structure year, and experimental method. Entries can be selected for in-depth review or submitted via an integrated feedback form. The lower panel presents the full record set with expert-verified transmembrane counts alongside predictor outputs, supporting real-time search by PDB code, classification, or transmembrane count.
2. Outlier Detection. This view supports topology and metadata assessment by combining a PCA projection with DBSCAN [36] clustering to distinguish inliers from outliers, a box plot of locality, spread, and skewness for selected attributes, and a scatter plot matrix (SPLOM) for cross-attribute outlier identification via brushing and linking. It is used to highlight unusual combinations of experimental conditions, resolutions, and topology descriptors in support of topology benchmarking and assessment. These views were evaluated in Tasks 2 and 3 of the user evaluation.
3. Single-Entry Structural. This record-centric view provides an integrated single-entry structural inspection interface that combines 3D molecular visualization with reconciled annotations, topology predictions, expert labels, and model outputs. Users can search by PDB code, UniProt, or OPM identifier. A metadata panel presents core annotations (accession, taxonomy, sequence), computed features (helices, strands, active sites, transmembrane segments), topology predictions, and expert- and machine learning (ML)-based classifications with confidence scores, together with scientific assessment flags indicating benchmark suitability and context-dependent cases.

Analytical visualizations were implemented using Altair [37] and Plotly [38] to produce reproducible, interactive linked views. 3D molecular visualization is provided through the Mol* [39] frontend structural viewer, which integrates entry-level structural rendering with topology and annotation overlays. Together, these views expose provenance-preserving integration, disagreement-aware reconciliation, topology-comparison workflows, and model-assisted annotations to end users in an interpretable, task-oriented form.

### Task-oriented user evaluation

We conducted a task-oriented evaluation to assess whether selected MetaMP views supported representative analytical workflows, focusing on the Summary Statistics and Outlier Detection views. Because MetaMP is an interactive analytical platform, usability is relevant to whether selected workflows can be operated reliably; however, this evaluation was not designed to measure biological discovery, scientific impact, or superiority over existing tools.

#### Hypotheses

Three hypotheses guided the quantitative analysis. For each, the null hypothesis states no effect; the alternative hypothesis states the expected directional or structural effect. H1 (Learning effectiveness): The null hypothesis is that completion time does not differ between the training and testing phases; the alternative hypothesis is that a structured training phase reduces completion time in the subsequent testing phase. H2 (Task difficulty): The null hypothesis is that completion time does not vary across tasks; the alternative hypothesis is that completion time differs significantly across tasks of differing complexity, irrespective of phase. H3 (Speed–accuracy trade-off): The null hypothesis is that completion time is not associated with response correctness; the alternative hypothesis is that users who complete tasks more quickly are more likely to produce incorrect responses. The H3 analysis is exploratory because the training phase included guided answers by design.

#### Participants and ethical considerations

All 24 participants (P1 to P24) were recruited by email from users with varying familiarity with biological databases and structural biology. Sociodemographic data collected included gender, years of experience, current status, and domain of expertise. Participation was voluntary; participants provided informed consent before beginning, and no directly identifying data were retained beyond self-reported role and experience level. The study was conducted in accordance with the ethical guidelines of the Centre for Artificial Intelligence at the Robert Koch Institute, Berlin, Germany.

#### Apparatus and measurements

The study was conducted entirely online within MetaMP using a custom-built integrated survey module covering onboarding, sociodemographic data collection, training, task execution, and usability evaluation. Task-level measurements included correctness, completion time, number of clicks, and optional qualitative comments.

Usability was assessed via an adapted post-study questionnaire. Items 5 to 14 reproduced the standard 10-item System Usability Scale (SUS), scored using the canonical SUS procedure [40]: $S_{i}=Q_{i}-1$ for positively worded items and $S_{i}=5-Q_{i}$ for negatively worded items, scaled to 0 to 100. Items 1 to 4 were study-specific and analyzed separately from the SUS total. SUS scores were interpreted using the adjective-rating guidance of Bangor et al. [25]. Records with missing SUS-item responses were excluded from score aggregation.

#### Tasks

Three consecutive tasks each comprised a training phase (with guidance, correct answers, and screenshots) followed by an independent testing phase with no time limit. A workflow tour familiarized participants with the platform before tasks began. The full question set is given in Table 5. These tasks were designed to evaluate interaction with representative analytical workflows rather than to measure specialist domain expertise in MP biology or expert curation performance. Task 1 assessed the Summary Statistics view for identifying temporal trends in structure-determination methods. Task 2 assessed outlier identification in grouped resolution data using box plots. Task 3 assessed whether DBSCAN-detected outliers matched those visible in the SPLOM via brushing and linking.

**Table 5.** Questions used in the task-based user evaluation. Each task followed a 2-step process: a training phase with guided answers, followed by a testing phase requiring independent responses.

Question	Type
**Task 1. Summary Statistics**	Training test
1. Which method appears to be most used?	
2. Which experimental method appears to be growing faster now?	
**Task 2. Outlier Identification**	Training test
3. Identify MP structure groups that contain outliers.	
4. Study the variations in resolution values using electron microscopy, focusing on the Monotopic group in the box plot. How many outliers are evident?	
**Task 3. Outlier Detection**	Training test
5. How many outliers in the SPLOM were not identified by MetaMP using DBSCAN?	
6. Do you observe any outliers in the SPLOM that MetaMP failed to detect using DBSCAN?	

#### Statistical analysis

H1 was tested using a Wilcoxon signed-rank test on paired training-versus-testing completion times. H2 was examined using a Friedman test, with a repeated-measures ANOVA as a sensitivity analysis. H3 was assessed using logistic regression with completion time as the independent variable and correctness as the dependent variable. Because training items included guidance by design, the H3 analysis is exploratory rather than a pure unguided-task benchmark. A clustered GEE model served as a sensitivity analysis to account for repeated responses per participant. Nonparametric tests were prioritized where normality assumptions were not met.

Usability scores were summarized descriptively using mean, median, standard deviation, 95% CI, and score distribution. Other descriptive user-study summaries reported in the Results were likewise calculated at the participant level with 95% CIs using the *t* distribution (*df* = 23). Because completion-time variables were right-skewed, their CIs should be interpreted as approximate descriptive summaries rather than strict normal-theory limits. Study-specific items and qualitative comments were analyzed separately from the SUS total to preserve the integrity of the canonical score.

#### Scope of the evaluation

The evaluation assessed usability of selected analytical workflows rather than the full platform. Findings should be interpreted as targeted evidence for task support in 2 representative views, not as a complete usability validation of MetaMP.

### Limitations

The current MetaMP release has 5 principal limitations. First, the expert-reviewed benchmark is intentionally focused rather than comprehensive. The 121-record reference set was assembled from cases in which MPstruc and OPM disagreed in broad-group classification, making it well suited for evaluating reconciliation under difficult conditions, but not for estimating annotation quality across the full MP landscape. It should therefore be interpreted as a targeted benchmark for disagreement handling rather than as a universal gold standard. Estimating performance across typical MP records will require a separate validation set that includes independently reviewed nondiscrepant entries.

Second, MetaMP integrates and compares annotations primarily at the PDB-entry level, whereas several topology predictors operate at the sequence or chain level. This difference in granularity is particularly relevant for multichain assemblies and structurally complex records, where entry-level and chain-level interpretations may diverge. Although MetaMP mitigates this through provenance preservation and caution flags, some cases still require explicit chain-resolved structural interpretation.

Third, the broad structural-group classifier is intended as an assistive model rather than a definitive annotation engine. Its target labels are standardized MPstruc broad-group annotations rather than independently adjudicated expert labels. Consequently, strict disagreement with the expert benchmark, particularly within the 121 records selected because MPstruc and OPM disagreed, should be interpreted as a limitation of the inherited training-label convention rather than as evidence that the model resolves contested annotations autonomously. Its performance also depends on the quantity and consistency of available labeled data. Moreover, the current semi-supervised workflow derives its withheld-label subset from the retained labeled training scope rather than from a large independent pool of naturally unlabeled records. The resulting models are therefore best used to support prioritization and expert review rather than fully automated classification.

Fourth, the expert benchmark labels were assigned by a single annotator. Although each assignment was supported by structural evidence, published topology data, and cross-source comparison, the single-annotator design introduces a risk of individual-specific bias in ambiguous cases. Users should therefore treat these labels as expert-informed reference assignments rather than as a formally adjudicated gold standard. Future development should include multi-annotator review for a subset of contested entries, with explicit recording of annotator count, agreement level, and unresolved sources of disagreement.

Fifth and last, MetaMP’s current coverage remains partly shaped by upstream database scope and curation. Because the harmonized release is anchored on MPstruc-linked entries, MPs absent from MPstruc may not yet appear as primary MetaMP records, even if related structures exist in the PDB. In addition, some apparent inconsistencies reflect source-specific release timing, identifier replacement history, or differing curation practices rather than locally resolvable annotation errors. Future development will therefore focus on expanding expert-reviewed coverage, extending integration to additional resources, and broadening external validation of the reconciliation framework. The task-oriented user evaluation should also be interpreted within scope. It assessed selected analytical workflows rather than the full platform and therefore provides targeted evidence for usability in representative views, not a complete validation of all MetaMP functions. The study did not include a head-to-head comparison with existing resources or expert-only domain-specific curation tasks.

## Discussion

MP annotations are distributed across resources that differ in scope, curation strategy, identifier stability, and structural interpretation. This fragmentation is not merely a data engineering inconvenience; it has direct consequences for the reliability of downstream computational analysis, benchmarking, and classification. MetaMP was developed to address this problem not by replacing primary databases but by making their overlaps, conflicts, and curation differences explicit, traceable, and actionable within a single provenance-aware framework.

This positions MetaMP differently from related resources. UniTmp integrates transmembrane-protein annotations across several established databases [19], and TMAlphaFold extends membrane-oriented interpretation to AlphaFold-derived structures [21]. MetaMP addresses a complementary but distinct problem: explicit cross-source reconciliation, review-state tracking, benchmark qualification, and expert-guided triage across experimentally anchored records. Its value lies not in breadth alone but in surfacing the inconsistencies that other integration efforts may absorb without making them explicit.

The rapid expansion of open predicted-structure resources amplifies rather than reduces this need for reconciliation. Recent large-scale releases such as ESMFold2 and ESM Atlas provide open access to billions of protein sequences and more than one billion predicted structures [41]. These resources substantially broaden the searchable protein-structure landscape, particularly for proteins outside intensively curated experimental datasets. However, they also increase the importance of explicitly distinguishing among experimentally resolved structures, predicted structures, source metadata, confidence estimates, and benchmark-eligible records. MetaMP is therefore best viewed as complementary infrastructure for tracking provenance, surfacing disagreement, and providing expert-guided qualification in an increasingly prediction-rich protein-data ecosystem.

A central insight motivating MetaMP’s design is that disagreement between curated resources is itself scientifically informative. Differences between MPstruc, OPM, expert review, and topology predictors are not always errors. They often reflect genuinely different curation objectives, taxonomic conventions, or levels of structural granularity. This is especially relevant for MPs, where topology and function can depend critically on oligomeric organization, insertion state, lipid environment, and assembly context. Certain structures can adopt different membrane-associated states depending on their environment, meaning that classification disagreement in such cases may reflect biological ambiguity rather than curation failure. By separating broad-group disagreement, transmembrane-count disagreement, and boundary-level disagreement, MetaMP makes annotation instability interpretable rather than invisible.

The expert-linked discrepancy benchmark offers a concrete illustration of this instability. Because the benchmark was intentionally assembled from cross-source disagreement cases, it should be interpreted as a stress test of reconciliation rather than as an estimate of disagreement prevalence across the full harmonized dataset. Although this subset represents a small fraction of the harmonized records, it is especially relevant for benchmark assembly, classifier evaluation, and curation triage because it concentrates unresolved annotation ambiguity rather than routine consensus cases. Benchmark or training datasets assembled from these resources without provenance checks or cross-source disagreement filtering may include contested records whose labels reflect unresolved biological, structural, or curation ambiguity. In such cases, a model can appear to make an error when it is instead reproducing one defensible source interpretation over another. Within that deliberately difficult subset, substantial discordance remained visible across source labels, expert review, and predictor outputs, reinforcing the need for a framework that preserves provenance and supports structured review rather than silent consensus.

Temporal analysis of the harmonized dataset further corroborates the well-documented advance in cryo-electron microscopy resolution [42–44]. Within the MetaMP dataset, cryo-EM has emerged as the dominant structure-determination method for MPs in recent years, surpassing x-ray crystallography in later periods. This trend is directly observable through MetaMP’s harmonized analytical views and illustrates how integrated structural metadata can support interpretable historical analysis.

The harmonization process also surfaced metadata-quality issues beyond classification disagreement. Several entries were identified as withdrawn, unreleased, or marked as withdrawn unreleased depositions, creating conflicts between deposition status and MP curation that are difficult to detect from within any single source database. More generally, identifier replacement history and asynchronous source updates contributed to forms of annotation instability that are often hidden during routine browsing but become visible once provenance is preserved systematically.

The disagreement observed between MPstruc and OPM also reflects a broader absence of community consensus on MP taxonomy. OPM organizes proteins hierarchically by type, class, superfamily, and family using structural information from SCOP and TCDB, while MPstruc uses a 3-group scheme based on membrane interaction mode [26,28]. These conventions are not arbitrary; they serve different analytical purposes and are both biologically defensible. MetaMP adopts the MPstruc 3-group scheme as its classification anchor while preserving OPM labels for comparison, making both perspectives available without forcing a single taxonomy on the user.

The transmembrane-segment benchmarking reinforces this picture of systematic variation. Within the current entry-level export, agreement varied substantially across sequence-based predictors, structure-linked methods, and annotation-derived segment counts, reflecting differences in predictor availability and overlap across the benchmark subset. Importantly, MetaMP does not use predictors to validate experimental records. Predictors serve as comparison signals that help identify entries where legacy annotations, expert labels, and computational expectations diverge, enabling a structured progression from broad disagreement statistics to concrete record-level review. Cases where model predictions and source labels diverged were treated as candidates for inspection rather than evidence of annotation error, since resolving such disagreements requires structural evidence beyond what automated comparison can provide.

The broad structural-group classifier contributes most when used in this same spirit of expert support. The presence of predictive signal in harmonized structural metadata confirms that broad MP grouping can be computationally assisted, but the classifier is a reproducible baseline, not a replacement for biological judgment. Its reliable use depends on the same provenance checking and discrepancy qualification that MetaMP applies when defining target labels, benchmark eligibility, and downstream evaluation sets. Its greatest practical value lies in prioritizing records for expert review and flagging cases where source annotations and model expectations do not align. Improving its reliability over time will require not only an expanded training set but also greater coordination among MP databases to reduce the annotation inconsistencies that propagate into model training.

Taken together, MetaMP’s analyses demonstrate that fragmented annotation is a structured, measurable phenomenon with predictable consequences for computational analysis and benchmarking. The platform’s central contribution is therefore not only data integration, but a framework for making those consequences visible, interpretable, and manageable. Future development should prioritize expanding the expert-reviewed benchmark, broadening database coverage to include resources such as PDBTM and UniTmp, and strengthening external validation so that the reconciliation framework becomes progressively more representative of the full MP structural landscape.

## Conclusion

MetaMP provides a provenance-aware reconciliation framework for MP annotation, integrating metadata from MPstruc, PDB, OPM, and UniProt into a unified and searchable resource. Its principal contribution is not the aggregation of data that are already publicly available, but the systematic surfacing of where those data agree, where they conflict, and where the conflict may warrant closer biological interpretation.

The analyses presented here show that cross-source disagreement is a measurable and structured pattern rather than isolated noise, and that this instability has direct consequences for downstream benchmarking, interpretation, and computational classification. By preserving provenance, exposing disagreement, qualifying benchmark suitability, and supporting expert-guided review, MetaMP provides a more realistic basis for working with MP annotations than a consensus-only integration model.

In practice, MetaMP is most valuable as an expert-support environment for identifying, contextualizing, and prioritizing contested MP records rather than automatically resolving every ambiguity. Future development should focus on expanding the expert-reviewed benchmark and broadening database coverage to include resources such as PDBTM and UniTmp, alongside stronger external validation to make the reconciliation framework progressively more representative of the structural landscape.

## Data Availability

All underlying MP records are publicly available from their respective primary sources: MPstruc (https://blanco.biomol.uci.edu/mpstruc/), RCSB PDB (https://www.rcsb.org/), OPM (https://opm.phar.umich.edu/), and UniProt (https://www.uniprot.org/). MPstruc source data were retrieved on 2026 March 27 and serve as the initial anchor for the harmonized dataset. The full harmonized MetaMP snapshot used for analysis in this paper, including all derived discrepancy fields, machine-learning outputs, and transmembrane-segment prediction results, reflects the live system state as of 2026 April 13. The interval between these 2 dates reflects the processing time required for incremental harmonization, topology-prediction pipeline execution, and internal validation after the initial source fetch. The MetaMP software is open-source and distributed under the MIT License. The complete source code, database schema, Docker configuration, and expert benchmark annotations are available at https://github.com/Ebenco36/MetaMP-Server. The repository includes a quick-start workflow that launches the full local web application from published Docker images using the documented command ./scripts/metamp-reviewer-start.sh; once running, the frontend is available locally at http://localhost/ and backend readiness can be checked at http://localhost:5400/api/v1/health/ready. The full dataset used in this research is embedded within the MetaMP application database. When the provided Docker container is instantiated, a fully populated PostgreSQL database is initialized containing the raw source tables (source_mpstruc, source_pdb, source_opm, and source_uniprot) as retrieved from the primary databases, the harmonized MetaMP table (membrane_proteins), and all derived annotation layers including discrepancy logs, machine-learning outputs, and transmembrane-segment predictions. Users may interact with the complete dataset through the MetaMP web interface or connect directly to the underlying PostgreSQL instance using any standard database client such as PGAdmin or psql; connection parameters and usage instructions are provided in the repository README.md.
