# Supplementary material for: MetaMP Ecosystem for Unified, Auditable, and Benchmark-Ready Data for Reliable Membrane Protein Annotation
Source: Comput Struct Biotechnol J. 2026 Aug 20;35(1):0165. doi: 10.34133/csbj.0165 (PMC13490743; doi:10.34133/csbj.0165)
Supplement: Supplementary file 1 [file csbj.0165.f1.pdf]

Supplementary Material.

MetaMP ecosystem for unified, auditable, and benchmark-ready data for reliable membrane protein annotation

Awotoro *et al.*

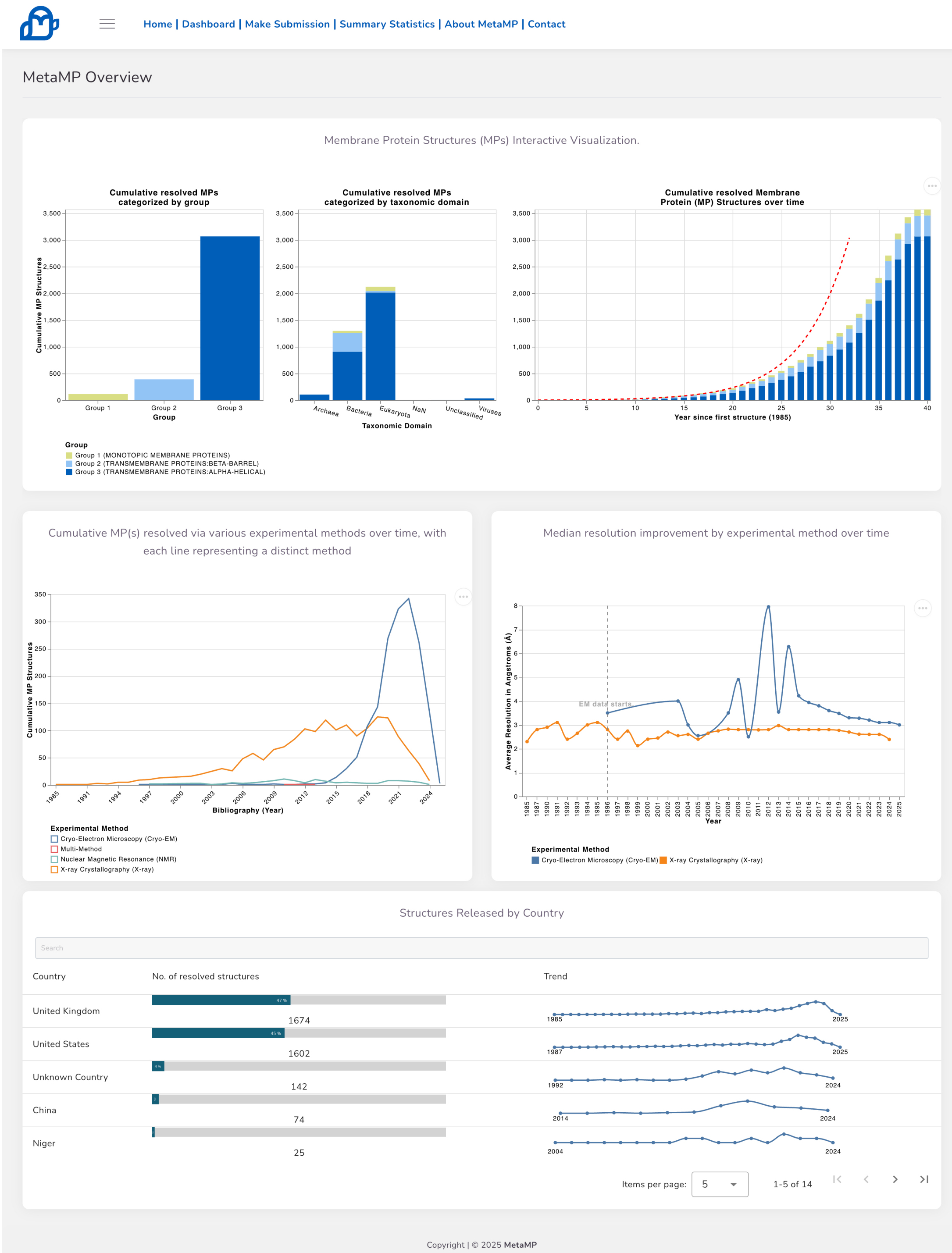

Figure 1. Overview
This view provides a detailed and interactive visualization of membrane protein (MP) structures. It categorizes cumulative MP data by groups, taxonomic domains, and historical trends, providing insights into structural progress and advancements in experimental methods, including median resolution improvements over time. The overview also presents country-specific contributions with trend analyses, serving as a comprehensive resource for researchers to explore MP datasets, identify patterns, and track technological milestones.

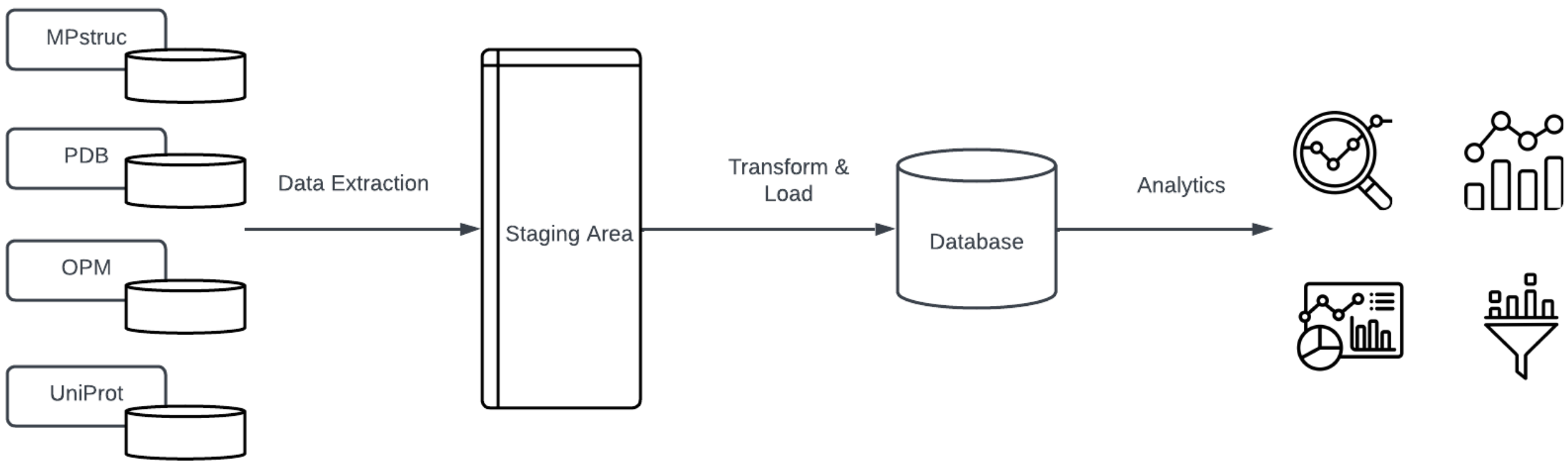

Figure 2. ETL workflow used by MetaMP. Data were extracted from MPstruc, PDB, OPM, and UniProt, processed in a staging layer, normalised into a harmonised schema, and loaded into the MetaMP database. The workflow preserves source-specific values and supports validation before release.

Table 1. MetaMP Updates to PDB Codes. This table shows the automatic update made to a set of PDB accession codes.

Old PDB code	New PDB code
5W7L	8G1N
3WXV	6KS0
3J8E	5TB0
3HGC	4NYK
4UPC	5A63
6FFV	8C7P
6AN7	6OIH
5TSI	5UAR
4J05	7SP5
3B8C	5KSD
4P6V	8ACY
1FUM	1L0V
3BZ1	4V62
3ARC	3WU2
3CJU	3EGV
5G1J	7PDC

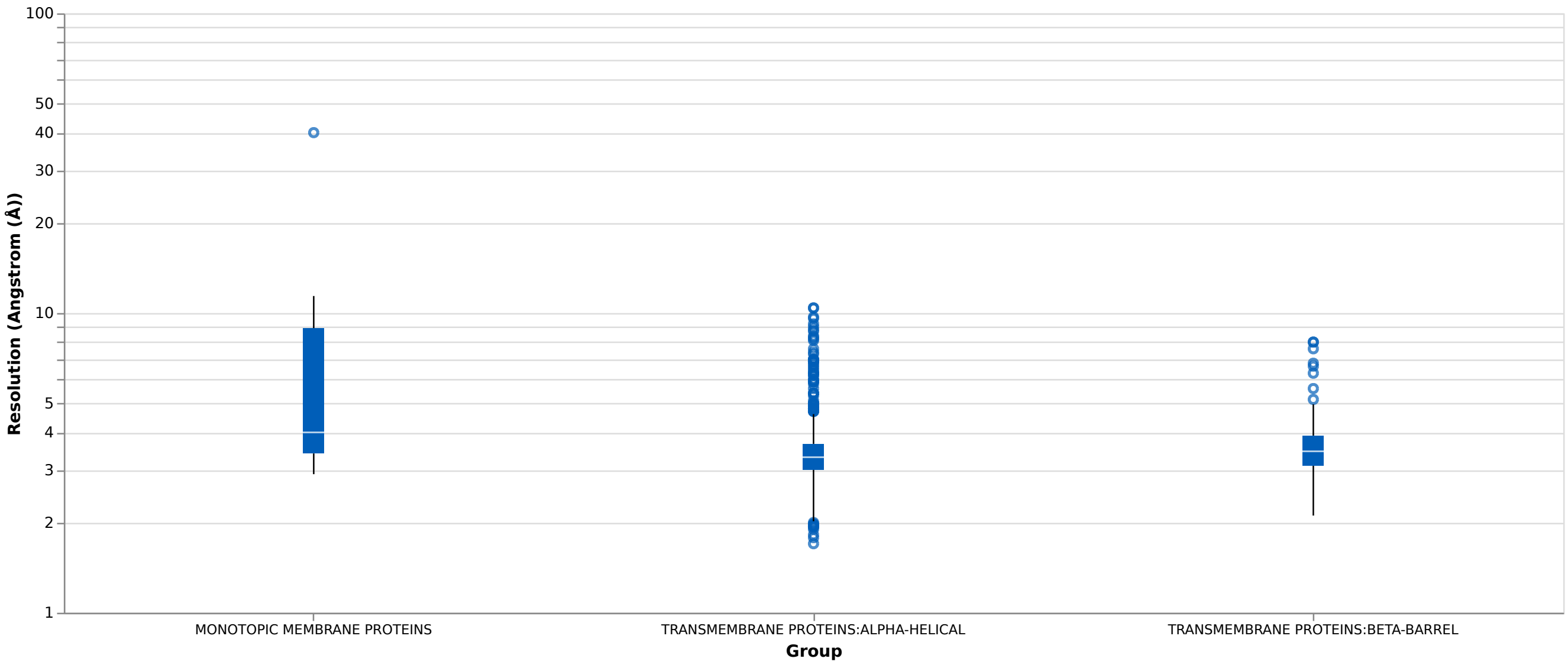

Figure 3. Outliers in Cryo-electron microscopy. The boxplot illustrates outliers in the Cryo-electron microscopy (EM) analysis. Notably, the protein coded 6ZG5 is identified as an outlier among MONOTOPIC MEMBRANE PROTEINS, displaying an unusually high resolution of 40 Ångströms (Å). This measurement markedly exceeds the typical resolution range observed in the dataset, highlighting the exceptional nature of this protein's resolution.

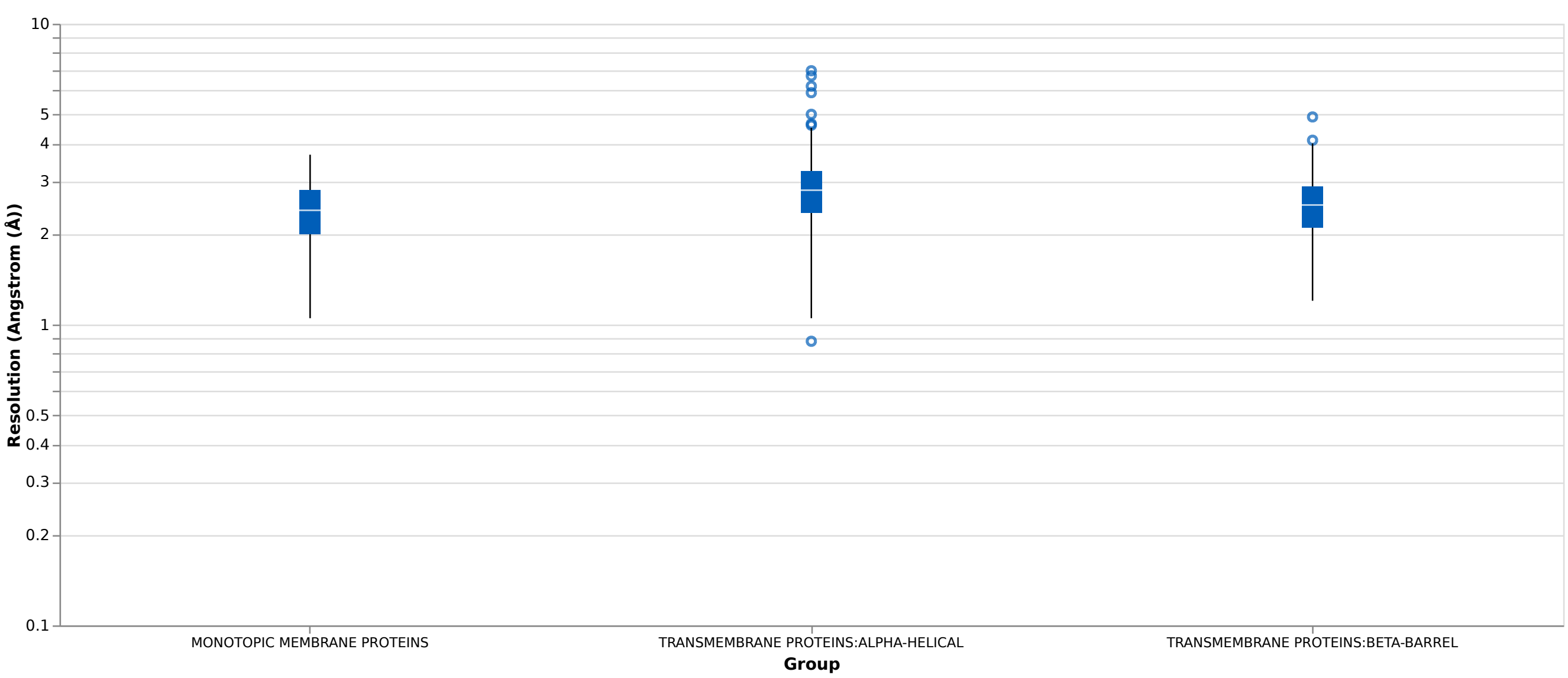

Figure 4. Outliers in X-ray crystallography. The boxplot shows the presence of numerous outliers in X-ray crystallography data for both TRANSMEMBRANE ALPHA-HELICAL and TRANSMEMBRANE BETA-BARREL proteins. The analysis highlights several instances where the resolution measurements significantly deviate from the typical range observed in these MP groups.

Table 2. Task metrics. Table showing the mean, median, and variance metrics for each task.

Task	Average Time (minutes) ± std	Median	Variance
1 (Training)	2.024 ± 2.364	1.176	5.588
2 (Testing)	1.111 ± 2.017	0.507	4.067
3 (Training)	0.682 ± 0.700	0.480	0.490
4 (Testing)	1.456 ± 2.389	0.821	5.707
5 (Training)	2.993 ± 2.960	2.317	8.761
6 (Testing)	1.083 ± 1.261	0.768	1.591

Table 3. Participant demographics. This table provides a comprehensive overview of the demographic characteristics of the participants involved in the study. It includes data on gender, years of experience in their respective domains, student status, and domain of expertise. Thirteen participants indicated that they are not students, while eleven indicated that they are students. In terms of domain expertise, approximately 46% (11 participants or ps) were from computer science, 29% (7 ps) from various other fields, 16% (4 ps) identified as bioinformaticians, 4% (1 participant) from biochemistry, and another 4% from structural biology. Additionally, the analysis indicated that approximately 71% (17 ps) had less than one year of experience, 8% (2 ps) had between one and three years, 8% (2 ps) had three to five years, 8 percent (2 ps) had five to ten years, and 8 percent (2 ps) had more than ten years of experience.

#	Gender	Years of Experience	Is Student	Domain
P1	♂	< 1	Yes	Computer Sci.
P2	♀	< 1	Yes	Bioinformatics
P3	♀	> 10	No	Structural Biology
P4	♀	< 1	Yes	Bioinformatics
P5	**NA**	< 1	No	Computer Sci.
P6	♂	< 1	Yes	Computer Sci.
P7	♂	5 – 10	No	Computer Sci.
P8	♀	< 1	Yes	Computer Sci.
P9	♂	< 1	No	Others
P10	♂	< 1	Yes	Computer Sci.
P11	♀	1 – 3	No	Others
P12	♂	3 – 5	No	Others
P13	♂	< 1	No	Computer Sci.
P14	♂	< 1	No	Bioinformatics
P15	♂	> 10	No	Biochemistry
P16	♂	< 1	No	Others
P17	♀	< 1	No	Bioinformatics
P18	♀	< 1	No	Computer Sci.
P19	♂	3 – 5	No	Computer Sci.
P20	♂	< 1	Yes	Computer Sci.
P21	♀	< 1	No	Others
P22	♀	1 – 3	No	Computer Sci.
P23	♀	< 1	Yes	Others
P24	♂	< 1	No	Others

Table 4. **The system usability scale (SUS) results: Usefulness**.

Description	Strongly Agree	Agree	Neutral	Disagree	Strongly Disagree
This tool would be useful for classifying membrane proteins into groups and sub-groups.	5	14	3	2	1
This tool would be useful for the study of membrane proteins.	5	15	3	2	0
Do you agree that this system accurately detects outliers based on the analysis of scatter plot matrix (SPLOM) ?	6	11	8	0	0
Do you agree that this system accurately detects outliers based on the analysis of the scatter plot (outlier detection plot) using DBSCAN ?	6	12	6	0	1
Do you agree that this system accurately detects outliers based on the analysis of the boxplot ?	4	14	6	0	1

Table 8. **Data discrepancy resolution**. Comparison between OPM database, MPstruc database, model predictions, and expert feedback. In the columns Group (OPM), Group (MPstruc) and Group (Predicted), three groups inherited from the MPstruc database are considered: Monotopic (1), transmembrane alpha (2), and transmembrane beta (3). The Group (Expert) proposes a fourth group Bitopic (4) to distinguish mono-, bi-, from poly-topic membrane proteins. A bitopic protein or single-pass membrane protein, also known as single-spanning protein, is a transmembrane protein that spans the lipid bilayer only once; in contrast to poly-topic membrane proteins. This distinction adds more granularity to the classes; hence, transmembrane alpha and beta are characterized as polytopic alpha-helical and polytopic beta-sheet proteins, respectively. Trans membrane (TM) segments are reported by the expert to support decision-making. (x) indicates the presence of subunits in the membrane protein. For example, (1x2) indicates a dimer with 1 TM per chain. (*) indicates no or not all TM present in the structure (**) indicates beta-barrel pore forming in its soluble form (no TM). This is specific to CDC pore forming family. The CDC pore-forming family: 1PFO, 3NSJ, 4HSC, 4CDB, 5IMW, 5IMY.

Year	PDB Code	Group (OPM)	Group (MPstruc)	Group (Predicted)	Group (Expert)	TM (Expert)
1997	1PFO	Monotopic membrane proteins	Transmembrane proteins:beta-barrel	Transmembrane proteins:beta-barrel	Transmembrane proteins:beta-barrel	0**
1997	1FDM	Bitopic proteins	Transmembrane proteins:alpha-helical	Transmembrane proteins:alpha-helical	Bitopic	1
1997	1AFO	Bitopic proteins	Transmembrane proteins:alpha-helical	Transmembrane proteins:alpha-helical	Bitopic	1
1998	2CPB	Bitopic proteins	Transmembrane proteins:alpha-helical	Transmembrane proteins:alpha-helical	Bitopic	1
1998	1B12	Transmembrane proteins:alpha-helical	Monotopic membrane proteins	Monotopic membrane proteins	Monotopic membrane proteins	
2002	1GOS	Bitopic proteins	Monotopic membrane proteins	Monotopic membrane proteins	Bitopic	1
2002	1MT5	Bitopic proteins	Monotopic membrane proteins	Monotopic membrane proteins	Monotopic membrane proteins	

Continued on next page

Table 8 – Continued from previous page

Year	PDB Code	Group (OPM)	Group (MPstruc)	Group (Predicted)	Group (Expert)	TM (Expert)
2002	1KN9	Transmembrane proteins:alpha-helical	Monotopic membrane proteins	Monotopic membrane proteins	Monotopic membrane proteins	
2003	1OJA	Bitopic proteins	Monotopic membrane proteins	Monotopic membrane proteins	Bitopic	1
2003	1MZT	Bitopic proteins	Transmembrane proteins:alpha-helical	Transmembrane proteins:beta-barrel	Bitopic	1
2004	1O5W	Bitopic proteins	Monotopic membrane proteins	Transmembrane proteins:alpha-helical	Bitopic	1
2004	1PJF	Bitopic proteins	Transmembrane proteins:alpha-helical	Transmembrane proteins:alpha-helical	Bitopic	1
2004	1UUM	Bitopic proteins	Monotopic membrane proteins	Monotopic membrane proteins	Bitopic	1*
2004	1T7D	Transmembrane proteins:alpha-helical	Monotopic membrane proteins	Monotopic membrane proteins	Monotopic membrane proteins	
2005	2BXR	Bitopic proteins	Monotopic membrane proteins	Transmembrane proteins:alpha-helical	Bitopic	1
2005	1YGM	Monotopic membrane proteins	Transmembrane proteins:alpha-helical	Transmembrane proteins:alpha-helical	Not a Membrane Protein	
2005	1ZLL	Bitopic proteins	Transmembrane proteins:alpha-helical	Transmembrane proteins:alpha-helical	Bitopic	1
2006	2GMH	Monotopic membrane proteins	Transmembrane proteins:alpha-helical	Transmembrane proteins:alpha-helical	Monotopic membrane proteins	
2006	2J58	Bitopic proteins	Transmembrane proteins:alpha-helical	Transmembrane proteins:beta-barrel	Bitopic	1x8
2006	2HAC	Bitopic proteins	Transmembrane proteins:alpha-helical	Transmembrane proteins:alpha-helical	Bitopic	1
2006	2J7A	Bitopic proteins	Transmembrane proteins:alpha-helical	Transmembrane proteins:alpha-helical	Bitopic	1x2
2007	2OLV	Bitopic proteins	Monotopic membrane proteins	Monotopic membrane proteins	Bitopic	1*
2007	2JO1	Bitopic proteins	Transmembrane proteins:alpha-helical	Transmembrane proteins:alpha-helical	Bitopic	1
2007	2OQO	Bitopic proteins	Monotopic membrane proteins	Monotopic membrane proteins	Bitopic	1*
2008	2QCU	Monotopic membrane proteins	Transmembrane proteins:alpha-helical	Monotopic membrane proteins	Monotopic membrane proteins	

Continued on next page

Table 8 – Continued from previous page

Year	PDB Code	Group (OPM)	Group (MPstruc)	Group (Predicted)	Group (Expert)	TM (Expert)
2008	2PRM	Bitopic proteins	Monotopic membrane proteins	Monotopic membrane proteins	Bitopic	1*
2008	2Z5X	Bitopic proteins	Monotopic membrane proteins	Transmembrane proteins:alpha-helical	Bitopic	1
2008	2VQG	Monotopic membrane proteins	Transmembrane proteins:alpha-helical	Transmembrane proteins:beta-barrel	Transmembrane proteins:alpha-helical	3
2008	2JWA	Bitopic proteins	Transmembrane proteins:alpha-helical	Transmembrane proteins:alpha-helical	Bitopic	1x2
2008	2K1L	Bitopic proteins	Transmembrane proteins:alpha-helical	Transmembrane proteins:alpha-helical	Bitopic	1x2
2008	3BKD	Bitopic proteins	Transmembrane proteins:alpha-helical	Transmembrane proteins:alpha-helical	Bitopic	1x4
2008	2RLF	Bitopic proteins	Transmembrane proteins:alpha-helical	Transmembrane proteins:alpha-helical	Bitopic	1x4
2009	3HYW	Monotopic membrane proteins	Transmembrane proteins:alpha-helical	Monotopic membrane proteins	Monotopic membrane proteins	
2009	3I65	Bitopic proteins	Monotopic membrane proteins	Monotopic membrane proteins	Bitopic	1*
2009	2KNC	Bitopic proteins	Transmembrane proteins:alpha-helical	Transmembrane proteins:alpha-helical	Bitopic	1
2009	3HD7	Bitopic proteins	Transmembrane proteins:alpha-helical	Transmembrane proteins:alpha-helical	Bitopic	1x2
2009	2KOG	Bitopic proteins	Transmembrane proteins:alpha-helical	Transmembrane proteins:alpha-helical	Bitopic	1x1
2009	2KIH	Bitopic proteins	Transmembrane proteins:alpha-helical	Transmembrane proteins:alpha-helical	Bitopic	1x4
2009	2KIX	Bitopic proteins	Transmembrane proteins:alpha-helical	Transmembrane proteins:alpha-helical	Bitopic	1x4
2009	3VMA	Bitopic proteins	Monotopic membrane proteins	Transmembrane proteins:alpha-helical	Bitopic	1
2009	3JQO	Transmembrane proteins:alpha-helical	Transmembrane proteins:beta-barrel	Transmembrane proteins:beta-barrel	Bitopic	1x14
2010	3NSJ	Monotopic membrane proteins	Transmembrane proteins:beta-barrel	Monotopic membrane proteins	Transmembrane proteins:beta-barrel	0**

Continued on next page

Table 8 – Continued from previous page

Year	PDB Code	Group (OPM)	Group (MPstruc)	Group (Predicted)	Group (Expert)	TM (Expert)
2010	2KSJ	Bitopic proteins	Transmembrane proteins:alpha-helical	Transmembrane proteins:alpha-helical	Bitopic	1
2010	2KS1	Bitopic proteins	Transmembrane proteins:alpha-helical	Transmembrane proteins:alpha-helical	Bitopic	1
2010	2K9Y	Bitopic proteins	Transmembrane proteins:alpha-helical	Transmembrane proteins:alpha-helical	Bitopic	1
2010	2L35	Bitopic proteins	Transmembrane proteins:alpha-helical	Transmembrane proteins:alpha-helical	Bitopic	1x3
2010	2L0J	Bitopic proteins	Transmembrane proteins:alpha-helical	Transmembrane proteins:alpha-helical	Bitopic	1x4
2010	3LBW	Bitopic proteins	Transmembrane proteins:alpha-helical	Transmembrane proteins:alpha-helical	Bitopic	1
2011	3ML3	Monotopic membrane proteins	Transmembrane proteins:beta-barrel	Transmembrane proteins:beta-barrel	Transmembrane proteins:beta-barrel	15*
2011	3PRW	Monotopic membrane proteins	Transmembrane proteins:beta-barrel	Transmembrane proteins:beta-barrel	Monotopic membrane proteins	
2011	3P1L	Monotopic membrane proteins	Transmembrane proteins:beta-barrel	Transmembrane proteins:beta-barrel	Monotopic membrane proteins	
2011	3Q7M	Monotopic membrane proteins	Transmembrane proteins:beta-barrel	Transmembrane proteins:beta-barrel	Monotopic membrane proteins	
2011	2YH3	Monotopic membrane proteins	Transmembrane proteins:beta-barrel	Transmembrane proteins:beta-barrel	Monotopic membrane proteins	
2011	3LIM	Monotopic membrane proteins	Transmembrane proteins:alpha-helical	Transmembrane proteins:beta-barrel	Bitopic	1
2011	2KPF	Bitopic proteins	Transmembrane proteins:alpha-helical	Transmembrane proteins:alpha-helical	Bitopic	1x2
2011	2L9U	Bitopic proteins	Transmembrane proteins:alpha-helical	Transmembrane proteins:alpha-helical	Bitopic	1x2
2011	2LJB	Bitopic proteins	Transmembrane proteins:alpha-helical	Transmembrane proteins:alpha-helical	Bitopic	1x4
2011	2KYV	Bitopic proteins	Transmembrane proteins:alpha-helical	Transmembrane proteins:alpha-helical	Bitopic	1x5
2012	3VMT	Bitopic proteins	Monotopic membrane proteins	Transmembrane proteins:alpha-helical	Bitopic	1

Continued on next page

Table 8 – Continued from previous page

Year	PDB Code	Group (OPM)	Group (MPstruc)	Group (Predicted)	Group (Expert)	TM (Expert)
2012	3Q54	Monotopic membrane proteins	Transmembrane proteins:beta-barrel	Transmembrane proteins:beta-barrel	Monotopic membrane proteins	
2012	2LCX	Bitopic proteins	Transmembrane proteins:alpha-helical	Transmembrane proteins:alpha-helical	Bitopic	1x2
2013	2LZL	Bitopic proteins	Transmembrane proteins:alpha-helical	Transmembrane proteins:alpha-helical	Bitopic	1x2
2013	2M8R	Bitopic proteins	Transmembrane proteins:alpha-helical	Transmembrane proteins:alpha-helical	Bitopic	1
2013	2LY0	Bitopic proteins	Transmembrane proteins:alpha-helical	Transmembrane proteins:alpha-helical	Bitopic	1x4
2013	2M3B	Bitopic proteins	Transmembrane proteins:alpha-helical	Transmembrane proteins:alpha-helical	Bitopic	1x5
2014	4LXJ	Bitopic proteins	Monotopic membrane proteins	Transmembrane proteins:alpha-helical	Bitopic	1
2014	4HSC	Monotopic membrane proteins	Transmembrane proteins:beta-barrel	Transmembrane proteins:beta-barrel	Transmembrane proteins:beta-barrel	0**
2014	4CDB	Monotopic membrane proteins	Transmembrane proteins:alpha-helical	Transmembrane proteins:beta-barrel	Transmembrane proteins:beta-barrel	0**
2014	2MFR	Bitopic proteins	Transmembrane proteins:alpha-helical	Transmembrane proteins:alpha-helical	Bitopic	1
2014	2M59	Bitopic proteins	Transmembrane proteins:alpha-helical	Transmembrane proteins:alpha-helical	Bitopic	1x2
2015	4TSY	Monotopic membrane proteins	Transmembrane proteins:alpha-helical	Transmembrane proteins:alpha-helical	Bitopic	1
2015	5EH4	Bitopic proteins	Transmembrane proteins:alpha-helical	Transmembrane proteins:alpha-helical	Bitopic	1x2
2015	4WOL	Bitopic proteins	Transmembrane proteins:alpha-helical	Transmembrane proteins:alpha-helical	Bitopic	1x3
2015	4QKC	Bitopic proteins	Transmembrane proteins:alpha-helical	Transmembrane proteins:alpha-helical	Bitopic	1x4
2016	5B49	Bitopic proteins	Monotopic membrane proteins	Monotopic membrane proteins	Monotopic membrane proteins	
2016	5IMW	Monotopic membrane proteins	Transmembrane proteins:beta-barrel	Transmembrane proteins:beta-barrel	Transmembrane proteins:beta-barrel	0**

Continued on next page

Table 8 – Continued from previous page

Year	PDB Code	Group (OPM)	Group (MPstruc)	Group (Predicted)	Group (Expert)	TM (Expert)
2016	5IMY	Monotopic membrane proteins	Transmembrane proteins:beta-barrel	Transmembrane proteins:beta-barrel	Transmembrane proteins:beta-barrel	0**
2016	2MIC	Bitopic proteins	Transmembrane proteins:alpha-helical	Transmembrane proteins:alpha-helical	Bitopic	1x2
2016	2N2A	Bitopic proteins	Transmembrane proteins:alpha-helical	Transmembrane proteins:alpha-helical	Bitopic	1x2
2016	5HK1	Bitopic proteins	Transmembrane proteins:alpha-helical	Transmembrane proteins:alpha-helical	Bitopic	1x3
2017	5LY6	Monotopic membrane proteins	Transmembrane proteins:beta-barrel	Transmembrane proteins:beta-barrel	Transmembrane proteins:beta-barrel	4
2017	5NUO	Transmembrane proteins:beta-barrel	Transmembrane proteins:alpha-helical	Transmembrane proteins:beta-barrel	Transmembrane proteins:beta-barrel	16x3
2017	5LV6	Bitopic proteins	Transmembrane proteins:alpha-helical	Transmembrane proteins:alpha-helical	Bitopic	1x2
2017	5JOO	Bitopic proteins	Transmembrane proteins:alpha-helical	Transmembrane proteins:alpha-helical	Bitopic	1x4
2018	6BFG	Bitopic proteins	Monotopic membrane proteins	Monotopic membrane proteins	Monotopic membrane proteins	
2018	6MLU	Transmembrane proteins:alpha-helical	Monotopic membrane proteins	Monotopic membrane proteins	Transmembrane proteins:alpha-helical	2
2018	6DLW	Monotopic membrane proteins	Transmembrane proteins:beta-barrel	Transmembrane proteins:beta-barrel	Transmembrane proteins:beta-barrel	88
2018	6H03	Monotopic membrane proteins	Transmembrane proteins:beta-barrel	Transmembrane proteins:beta-barrel	Transmembrane proteins:beta-barrel	72
2018	6F2D	Transmembrane proteins:alpha-helical	Transmembrane proteins:beta-barrel	Transmembrane proteins:alpha-helical	Transmembrane proteins:alpha-helical	34
2018	6HJR	Bitopic proteins	Transmembrane proteins:alpha-helical	Transmembrane proteins:alpha-helical	Bitopic	1x3
2018	6E10	Bitopic proteins	Transmembrane proteins:alpha-helical	Transmembrane proteins:alpha-helical	Bitopic	1x7
2018	6BKK	Bitopic proteins	Transmembrane proteins:alpha-helical	Transmembrane proteins:alpha-helical	Bitopic	1x4
2019	6NYF	Monotopic membrane proteins	Transmembrane proteins:alpha-helical	Transmembrane proteins:alpha-helical	Transmembrane proteins:beta-barrel	12*

Continued on next page

Table 8 – Continued from previous page

Year	PDB Code	Group (OPM)	Group (MPstruc)	Group (Predicted)	Group (Expert)	TM (Expert)
2019	6MQU	Bitopic proteins	Transmembrane proteins:alpha-helical	Transmembrane proteins:alpha-helical	Bitopic	1x5
2019	6JXR	Bitopic proteins	Transmembrane proteins:alpha-helical	Transmembrane proteins:alpha-helical	Bitopic	1x8
2019	6MTI	Monotopic membrane proteins	Transmembrane proteins:alpha-helical	Monotopic membrane proteins	Bitopic	1*
2019	6MJH	Bitopic proteins	Transmembrane proteins:alpha-helical	Transmembrane proteins:alpha-helical	Bitopic	1x4
2020	6S3S	Transmembrane proteins:alpha-helical	Transmembrane proteins:beta-barrel	Transmembrane proteins:alpha-helical	Transmembrane proteins:alpha-helical	34
2020	6S3R	Transmembrane proteins:alpha-helical	Transmembrane proteins:beta-barrel	Transmembrane proteins:alpha-helical	Transmembrane proteins:alpha-helical	36
2020	7K7A	Bitopic proteins	Transmembrane proteins:alpha-helical	Transmembrane proteins:alpha-helical	Bitopic	1x3
2020	7BV6	Bitopic proteins	Transmembrane proteins:alpha-helical	Monotopic membrane proteins	Bitopic	1*
2020	6NV1	Bitopic proteins	Transmembrane proteins:alpha-helical	Transmembrane proteins:alpha-helical	Bitopic	1x4
2020	6PVR	Bitopic proteins	Transmembrane proteins:alpha-helical	Transmembrane proteins:alpha-helical	Bitopic	1x4
2020	7K3G	Bitopic proteins	Transmembrane proteins:alpha-helical	Transmembrane proteins:alpha-helical	Bitopic	1x5
2020	6Z0G	Bitopic proteins	Transmembrane proteins:alpha-helical	Monotopic membrane proteins	Bitopic	1
2021	7OFM	Bitopic proteins	Monotopic membrane proteins	Transmembrane proteins:alpha-helical	Bitopic	1
2021	7AGX	Transmembrane proteins:alpha-helical	Transmembrane proteins:beta-barrel	Transmembrane proteins:alpha-helical	Transmembrane proteins:alpha-helical	34
2021	7OKN	Transmembrane proteins:alpha-helical	Transmembrane proteins:beta-barrel	Transmembrane proteins:beta-barrel	Bitopic	1x17
2021	7KN0	Bitopic proteins	Transmembrane proteins:alpha-helical	Transmembrane proteins:alpha-helical	Bitopic	1x2
2021	7LQ6	Bitopic proteins	Monotopic membrane proteins	Transmembrane proteins:alpha-helical	Bitopic	1

Continued on next page

Table 8 – Continued from previous page

Year	PDB Code	Group (OPM)	Group (MPstruc)	Group (Predicted)	Group (Expert)	TM (Expert)
2021	6LKD	Bitopic proteins	Transmembrane proteins:alpha-helical	Transmembrane proteins:alpha-helical	Bitopic	1x2
2022	7RSL	Transmembrane proteins:alpha-helical	Monotopic membrane proteins	Transmembrane proteins:alpha-helical	Transmembrane proteins:alpha-helical	2
2022	8A1D	Monotopic membrane proteins	Transmembrane proteins:beta-barrel	Transmembrane proteins:alpha-helical	Transmembrane proteins:beta-barrel	64
2022	7WSO	Bitopic proteins	Transmembrane proteins:alpha-helical	Transmembrane proteins:alpha-helical	Bitopic	1x4
2022	7XT6	Bitopic proteins	Transmembrane proteins:alpha-helical	Transmembrane proteins:alpha-helical	Bitopic	1x4
2022	7XQ8	Bitopic proteins	Transmembrane proteins:alpha-helical	Transmembrane proteins:alpha-helical	Bitopic	1x4
2022	7W2B	Bitopic proteins	Transmembrane proteins:alpha-helical	Transmembrane proteins:alpha-helical	Bitopic	1x3
2022	7FJD	Bitopic proteins	Transmembrane proteins:alpha-helical	Transmembrane proteins:alpha-helical	Bitopic	1x8
2022	7VU5	Bitopic proteins	Transmembrane proteins:alpha-helical	Transmembrane proteins:alpha-helical	Bitopic	1x2
2022	7MPA	Bitopic proteins	Transmembrane proteins:alpha-helical	Transmembrane proteins:alpha-helical	Bitopic	1
2023	8GI1	Bitopic proteins	Transmembrane proteins:beta-barrel	Monotopic membrane proteins	Bitopic	1x6

Table 5. The system usability scale (SUS) results: Intuitiveness. P7 and P20 highlighted that "*everything looks well-developed and quite straightforward to follow*" and that the questionnaire was "*extremely user-friendly with a visually appealing design.*" Although the majority of participants found the system to be generally intuitive and well-designed, an issue was noted about the task instructions by only one participant. P4 reported problems related to "*resetting the page and selecting/deselecting clusters*", which are functionalities covered in the provided instructions. This suggests that not all participants may have carefully read with the available instructions. This may indicate a need for shorter instructions or clearer instructional cues to guide participants.

Description	Strongly Agree	Agree	Neutral	Disagree	Strongly Disagree
I think I would like to use this system frequently and most people would learn to use it very quickly.	4	9	9	2	1
The system is intuitive, facilitating easy navigation to locate desired information.	6	11	4	3	1
I found the system unnecessarily complex and very cumbersome to use.	1	2	6	10	6
I would need the support of a technical person to use this system.	1	5	2	9	8
I thought there was too much inconsistency in this system.	0	3	3	14	5
I felt confident using the system and am very satisfied with the overall user experience.	3	12	6	3	1
The system loads quickly.	12	13	0	0	0

Table 6. The system usability scale (SUS) results: Design choices.

Description	Strongly Agree	Agree	Neutral	Disagree	Strongly Disagree
I found it easy to interact with the charts, identify outliers using the box-plot, and use the interactive elements.	7	11	5	2	0
The layout and organization of graphical elements are intuitive.	9	9	6	1	0

Table 7. Descriptive summaries for the task-oriented user evaluation. Reported values are participant-level means with standard deviations and 95% confidence intervals. Performance score is the count of correct responses across all six task questions, scored on a 0–6 scale. Completion-time summaries are reported in minutes.

Measure	Mean $\pm$ SD	95% CI	Unit / scale
Performance score	4.21 ± 0.98	3.80–4.62	0–6
Combined training + testing completion time	9.35 ± 8.63	5.70–12.99	minutes
Training-phase completion time	1.90 ± 1.74	1.16–2.64	minutes per task
Testing-phase completion time	1.22 ± 1.38	0.63–1.80	minutes per task
Task 1 total completion time	3.13 ± 3.30	1.74–4.53	minutes
Task 2 total completion time	2.14 ± 2.90	0.92–3.36	minutes
Task 3 total completion time	4.08 ± 3.98	2.40–5.76	minutes
SUS score	72.81 ± 15.77	66.16–79.47	0–100

Table 9. Global SHAP ranking for the selected semi-supervised *No-DR* Decision Tree bundle. Values are mean absolute SHAP magnitudes computed from a 500-row sample drawn from the eligible training matrix after benchmark exclusions.

Feature	Mean absolute SHAP
Membrane Thickness	0.0881
Membrane Topology Out	0.0275
Subunit TM Segments	0.0133
Membrane Topology In	0.00812
Gibbs Free Energy	0.00173
Helix Tilt	0.00111
Topology Subunit Count	0.000408

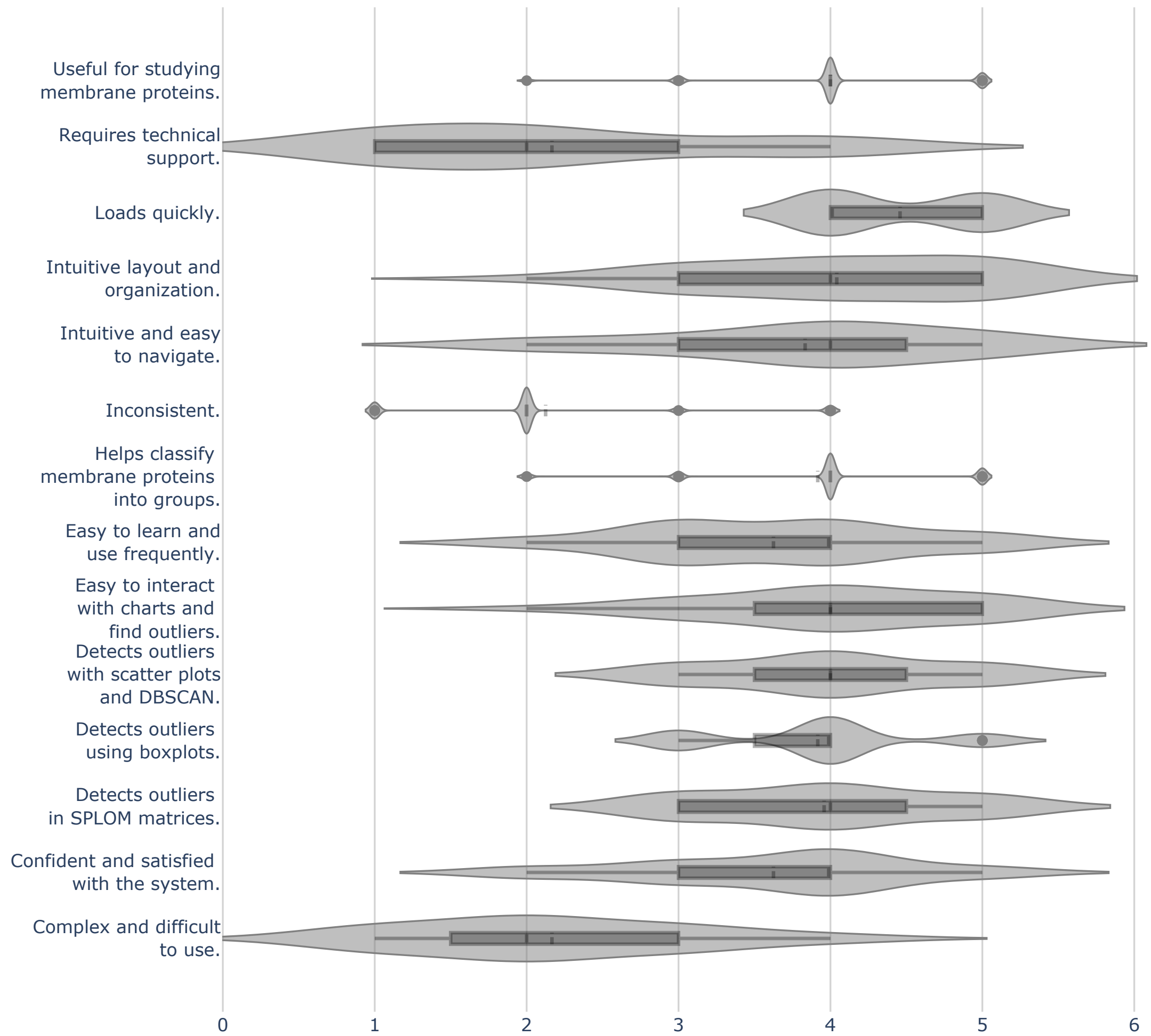

Figure 5. System Usability Scale (SUS) scores. The majority of participants evaluated the system as highly usable, well integrated, and loads quickly. P7 noted, "*All in all, it's a great system, fast and reliable.*" The integration of different visualization techniques was recognized as valuable and contributed to an overall positive user experience. In addition, during Task 3, some participants found the process of comparing outlier detection between SPLOM and the scatterplot DBSCAN (Density-Based Spatial Clustering of Applications with Noise) complex. P4 commented, "*Comparing outlier detection between SPLOM and DBSCAN by manually hovering over all data points seems unnecessarily complicated.*" To remedy this, we have changed the layout of the graphs for intuitive visual linking of the relevant visualizations, reduced the need for scrolling, and improved the interactive idiom of brushing and linking. Another criticism was that it was difficult to interpret certain graphs, especially those related to count data. P7 noted, "*The different aspect of the experiment was not really showing on the graph for easy interpretation on only 1 part. It was the graph relating to the count. You can see the count but unable to see which experiment is in blue or red when hovered, making it not too clear for the user.*" In this version, we have updated the most evident graphical marks that were superimposed on one another by changing the visual channel encodings. Furthermore, we are currently adding annotations with the objective of enhancing clarity. The feedback on the interactive features revealed both positive aspects and potential areas for further enhancement. For instance, one participant (P9) requested to improve a Drop List Field-related interaction and make the Apply Change button "*more visually prominent*". We made this adjustment according to the feedback. Another instance was a request from a couple of participants to improve the real-time brushing and linking during Task 3. Outlier Detection: "*When selecting data points in the SPLOM, the corresponding points should be dynamically displayed in the DBSCAN clustering scatterplot.*". We have addressed this feature request by making interactivity bi-directional across charts. This update improved the intuitiveness with which users can find and analyze outliers.

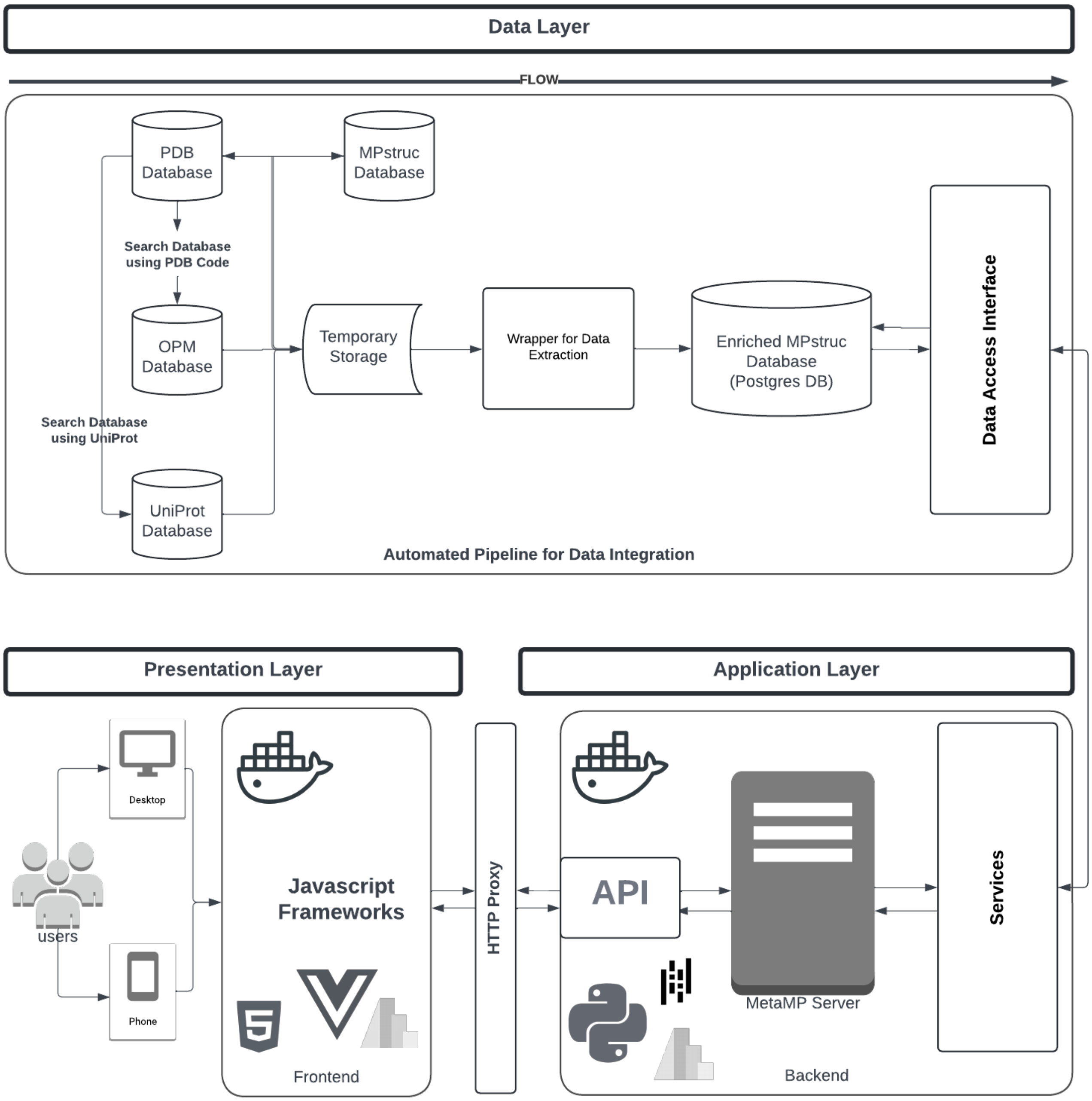

Figure 6. The three-tiered architecture of MetaMP. It comprises the Data, Application, and Presentation layers. The Data Layer integrates various experimental and computational sources. The Application Layer handles data processing and analysis. The Presentation Layer provides visualization and user interaction. This structure ensures robust, scalable, and comprehensive meta-analysis capabilities.

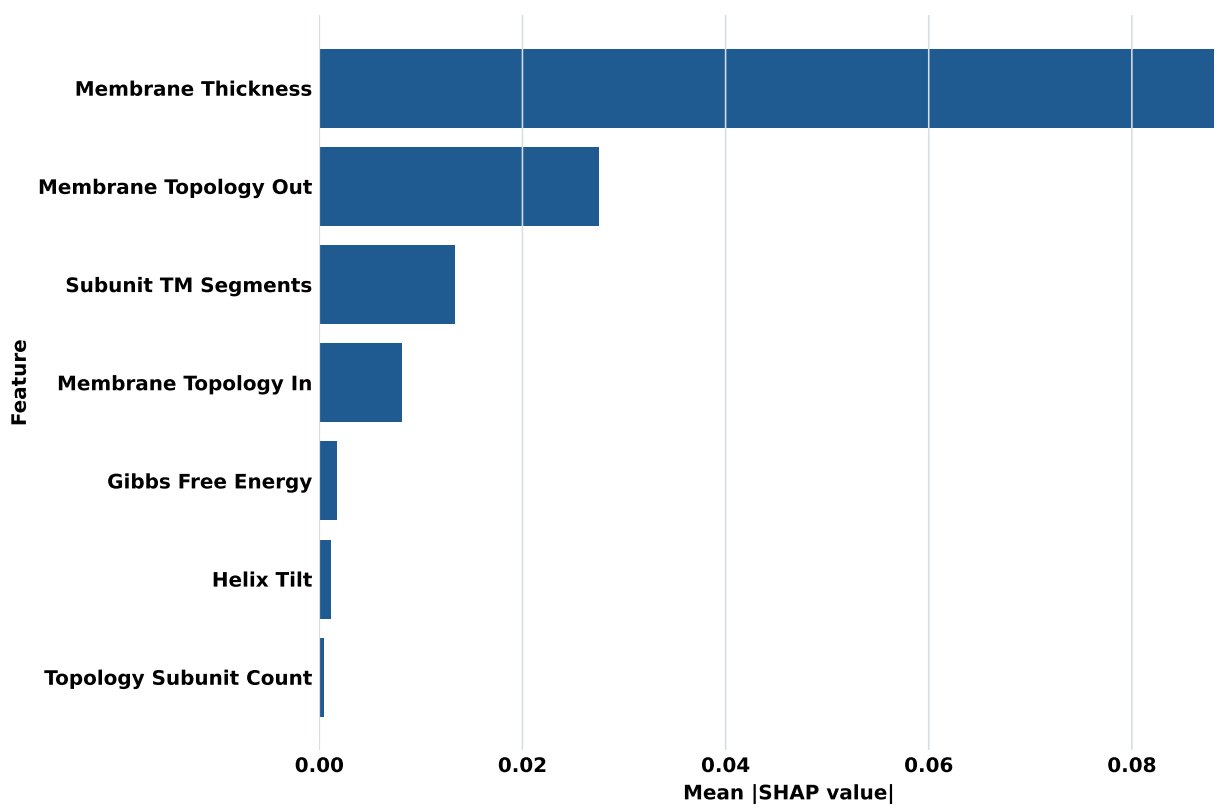

(a) Global SHAP ranking.

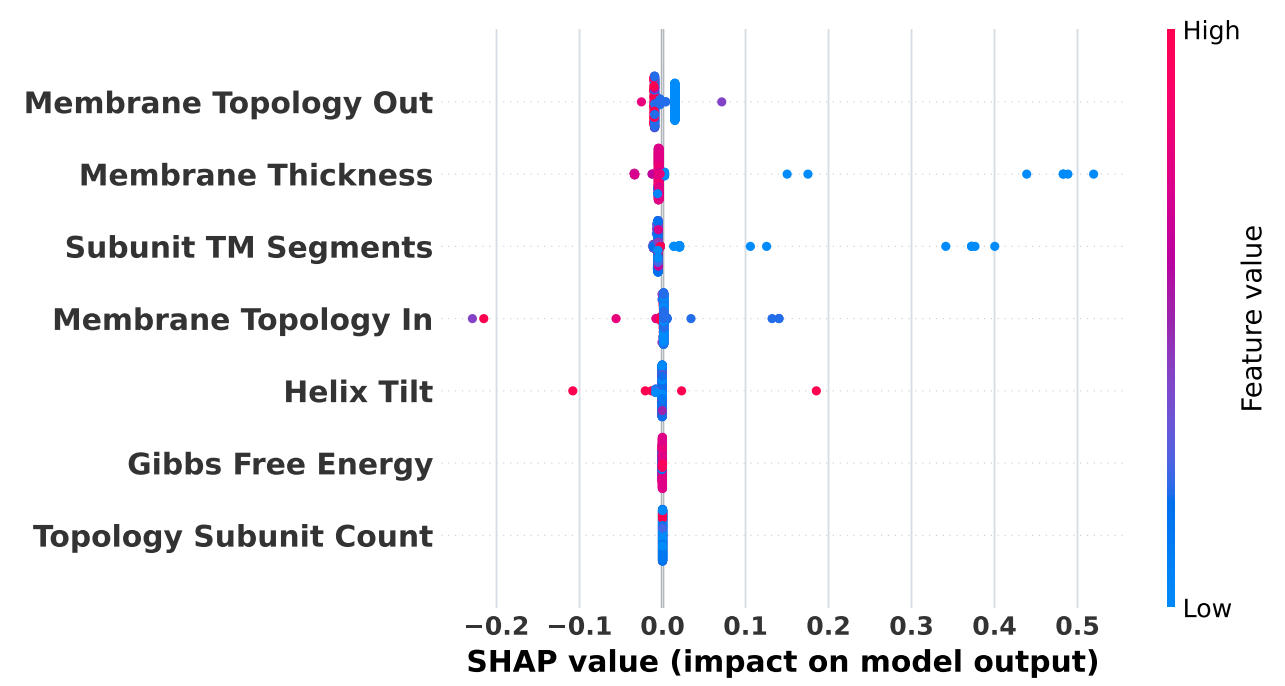

(b) Monotopic membrane proteins.

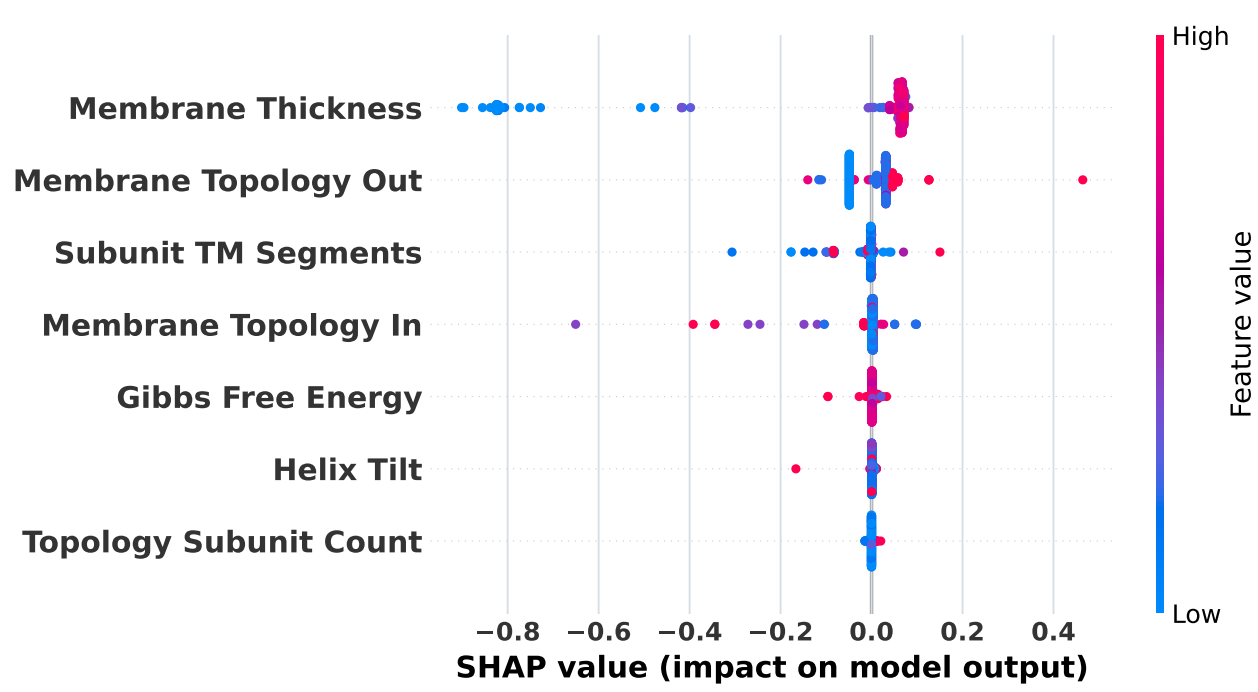

(c) Alpha-helical transmembrane proteins.

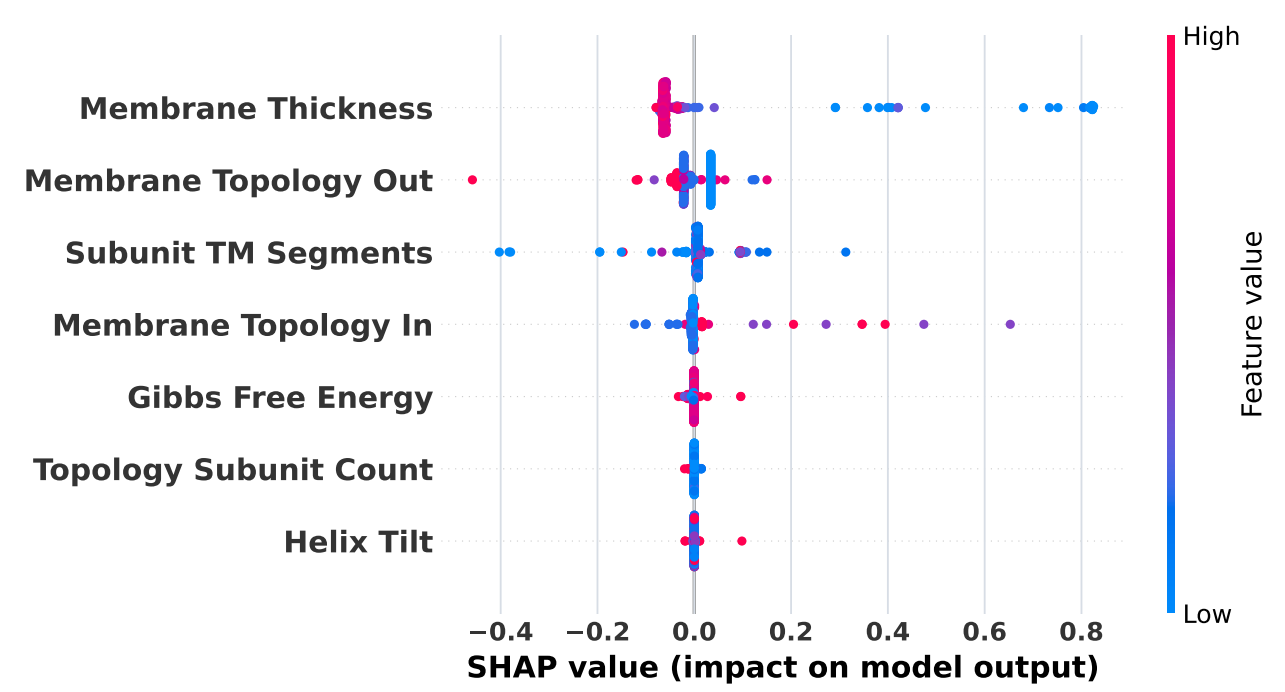

(d) Beta-barrel transmembrane proteins.

Figure 7. SHAP summaries for the selected semi-supervised *No-DR* Decision Tree production bundle. The top-left panel shows the global mean absolute SHAP ranking, and the remaining panels show class-specific beeswarm plots for monotopic, alpha-helical transmembrane, and beta-barrel transmembrane predictions.

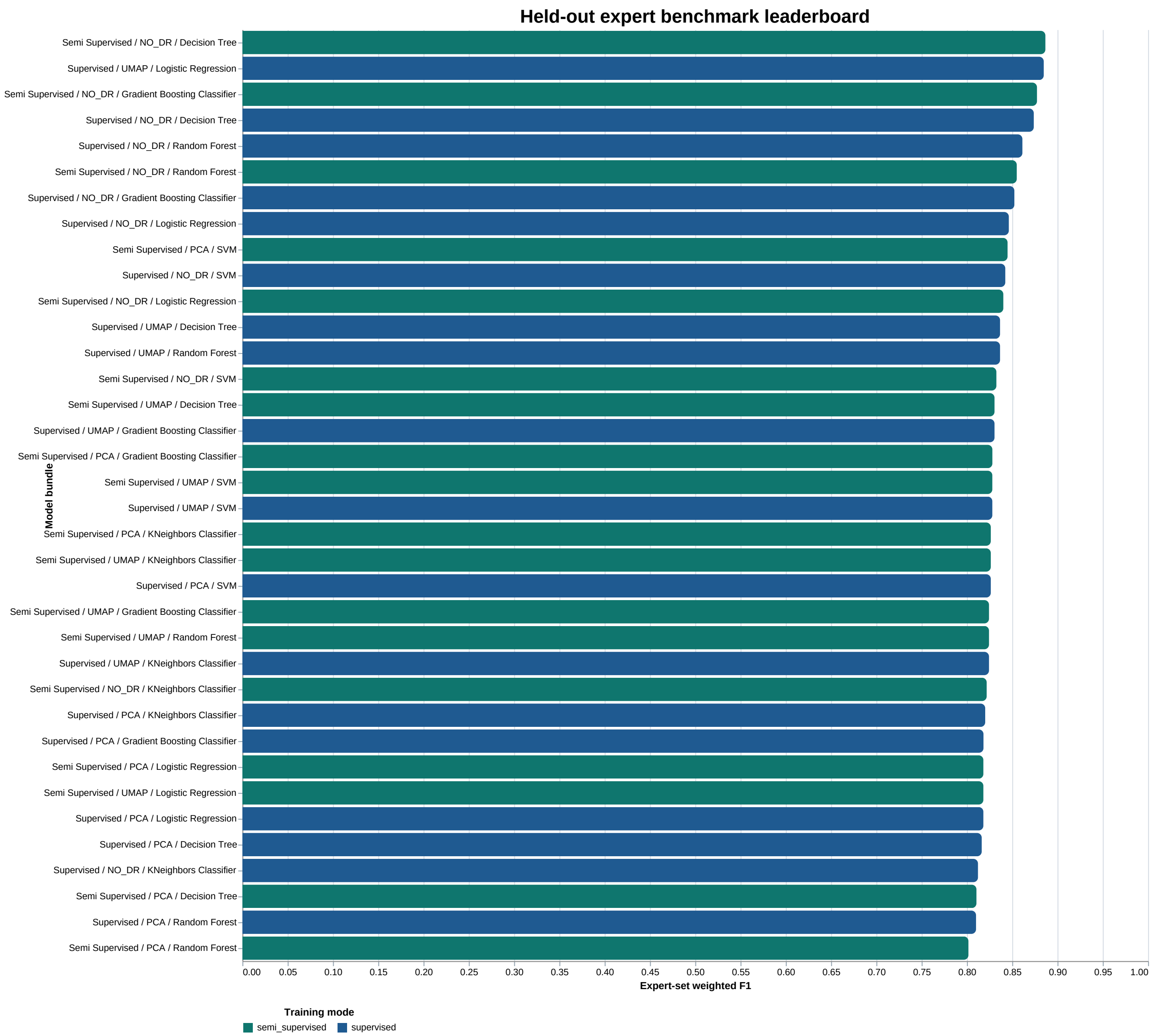

(a) Expert-benchmark leaderboard across evaluated bundles.

Figure 8. Summary views of production bundle selection. The semi-supervised *No-DR* Decision Tree bundle was selected for upload because it achieved the strongest held-out expert-benchmark performance, despite not having the numerically highest internal weighted F1.

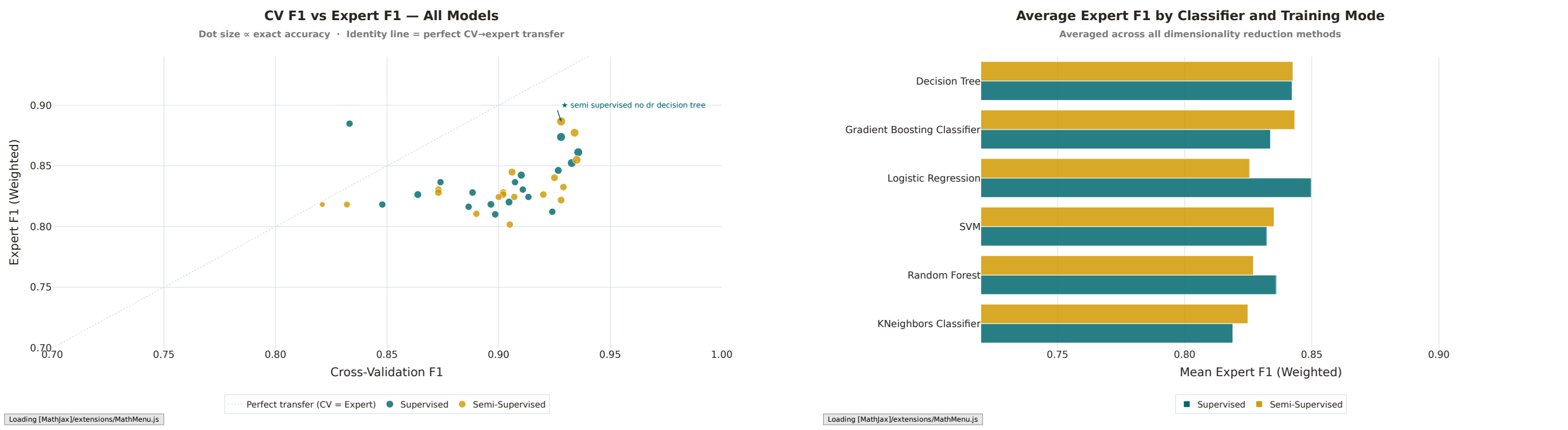

(a) Internal versus expert weighted F1.

(b) Classifier-family performance by training mode.

Figure 9. Primary model-registry summaries for the broad structural-group classifier. The left panel shows the relationship between internal and expert-set weighted F1 across all 36 evaluated bundles. The right panel shows classifier-family performance under supervised and semi-supervised training modes.

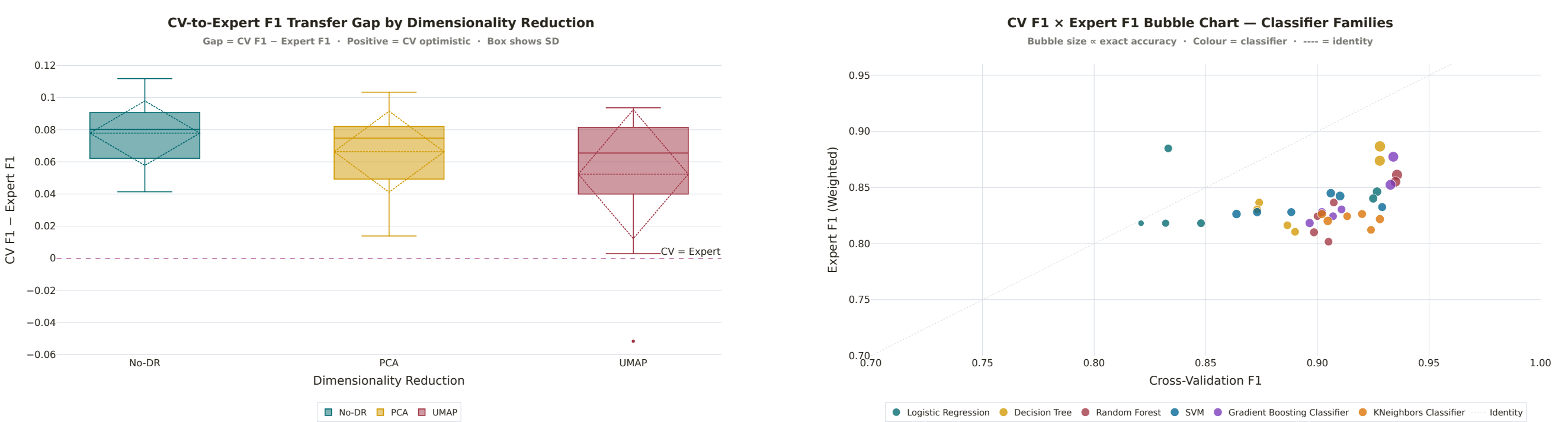

(a) Distribution of internal-to-expert performance gaps.

(b) Internal versus expert performance with exact-accuracy scaling.

Figure 10. Secondary model-registry summaries for the broad structural-group classifier. Together with Figure 9, these panels show that the strongest production bundles were not necessarily those with the highest internal score, and that *No-DR* bundles consistently occupied the upper tier of the expert-benchmark ranking.

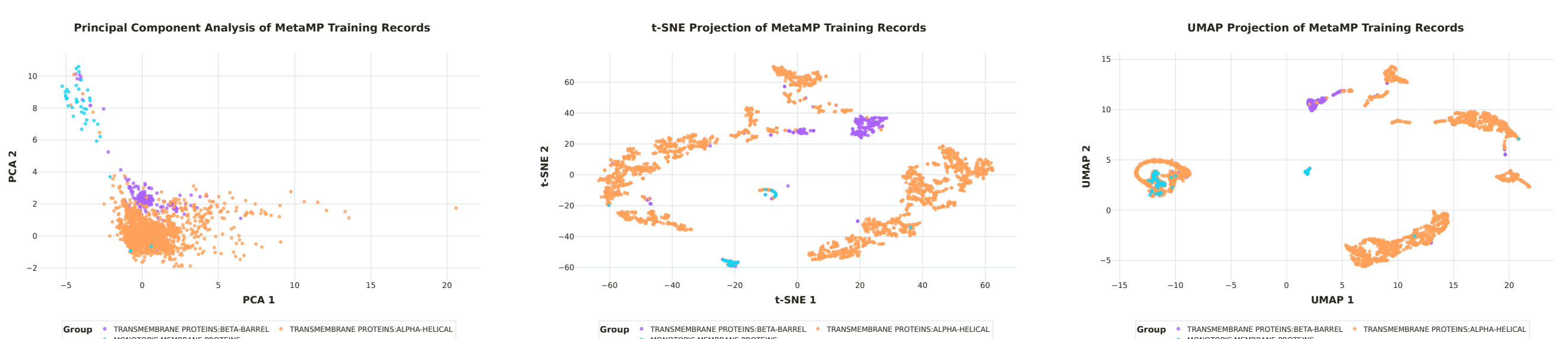

(a) PCA.

(b) t-SNE.

(c) UMAP.

Figure 11. Exploratory dimensionality-reduction projections of the production training matrix after benchmark exclusions. These projections were generated for visual inspection of the retained seven-feature OPM-derived training space and were not themselves used as the benchmark for final bundle selection beyond the production PCA and UMAP model views.
